# Diet-Morphology Correlations in the Radiation of South American Geophagine Cichlids (Perciformes: Cichlidae: Cichlinae)

**DOI:** 10.1371/journal.pone.0033997

**Published:** 2012-04-02

**Authors:** Hernán López-Fernández, Kirk O. Winemiller, Carmen Montaña, Rodney L. Honeycutt

**Affiliations:** 1 Department of Natural History, Royal Ontario Museum, Toronto, Ontario, Canada; 2 Department of Ecology and Evolutionary Biology, University of Toronto, Toronto, Ontario, Canada; 3 Section of Ecology, Evolution and Systematics, Department of Wildlife and Fisheries Sciences, Texas A&M University, College Station, Texas, United States of America; 4 Natural Science Division, Pepperdine University, Malibu, California, United States of America; Brigham Young University, United States of America

## Abstract

Genera within the South American cichlid tribe Geophagini display specialized feeding and reproductive strategies, with some taxa specialized for both substrate-sifting and mouth brooding. Several lineages within the clade also possess an epibranchial lobe (EBL), a unique pharyngeal structure that has been proposed to have a function in feeding and/or mouth brooding. A recently published genus-level phylogeny of Neotropical cichlids was used as the evolutionary framework for investigating the evolution of morphological features presumably correlated with diet and mouth brooding in the tribe Geophagini. We tested for possible associations between the geophagine epibranchial lobe and benthic feeding and mouth brooding. We also addressed whether the EBL may be associated with unique patterns of diversification in certain geophagine clades. Tests of binary character correlations revealed the EBL was significantly associated with mouth brooding. We also tested for a relationship between diet and morphology. We analyzed stomach contents and morphometric variation among 21 species, with data for two additional species obtained from the literature. Principal Components Analysis revealed axes of morphological variation significantly correlated with piscivory and benthivory, and both morphology and diet were significantly associated with phylogeny. These results suggest that the EBL could be an adaptation for either feeding or mouth brooding. The EBL, however, was not associated with species richness or accelerated rates of phyletic diversification.

## Introduction

The Neotropical region of South and Central America contains the largest diversity of freshwater fishes on earth, estimated at well over 7000 species [1]. Recent studies encompassing regional perspectives in phylogenetics, biogeography, geology, paleontology, and biodiversity have revealed that the evolution of the Neotropical freshwater fish fauna is the outcome of highly complex historical and ecological circumstances occurring over a period of tens of millions of years [2]–[5]. Of fundamental importance in understanding the components of such a complex history is clarifying the relative role of adaptive and non-adaptive processes in the diversification of Neotropical fish diversity. A viable approach to address this question is analysis of entire clades of so-called “incumbent” taxa, i.e. those which originated in the Neotropics and became the dominant faunas that today dominate its environments [6], [7].

Being the third most diverse family of Neotropical freshwater fishes after Characidae and Loricariidae [8], cichlids are an ideal group to study potentially adaptive processes in the evolution of Neotropical fishes. Cichlids are geographically widespread and sufficiently diverse to represent a good portion of the Neotropical diversity, yet manageable with respect to estimating phylogenetic relationships [9]–[16]. Altogether, cichlids represent an ideal system for both testing hypotheses of adaptation and exploring the processes responsible for diversification of the Neotropical freshwater fish fauna.

Several clades of Neotropical cichlid fishes reveal patterns of phylogenetic diversification and ecomorphological specialization [13]–[17]. For instance, the South American tribe Geophagini represents one of the most diverse groups of Neotropical cichlids, containing more than 300 species in 17 putative genera. Genera in this tribe form a monophyletic group possibly characterized by relatively rapid diversification as evidenced by the short internodes at the base of the geophagine tree [14], [16]. Genera also show a variety of specialized feeding and reproductive strategies, both of which are strongly reflected in their morphology. In contrast, intrageneric morphological and ecological variation appears to be much more limited [14]. With the exception of *Crenicichla*, a genus dominated by piscivorous species, many genera of Geophagini perform substrate sifting to obtain benthic invertebrates, a behavior that involves ingesting sandy or silty substrate and filtering out edible particles by means of a relatively stereotypical behavior known as “winnowing” [18]. Winnowing involves use of the oral jaws and the pharyngeal basket, and its functional morphology has been well studied in the family Embiotocidae [18]–[20]. Given the uniformity in the anatomy of the pharyngeal jaw apparatus (PJA) of cichlids and embiotocids and their presumed phylogenetic relatedness [21], [22], it can be assumed that winnowing in cichlids is performed in a similar manner [23]. In addition to substrate sifting through “winnowing,” several geophagine taxa perform mouth brooding.

Two clades of substrate-sifting geophagines [16] possess an epibranchial lobe (EBL), an antero-ventral expansion of the first epibranchial bone capped with cartilage and lined with pad-like gill rakers. The EBL has been hypothesized to be either an adaptation for mouth brooding [24] or for sifting of substrate and food particles [25]. Although other cichlid genera (e.g. *Retroculus*) have modifications of the first epibranchial bone [15], [26], the EBL is a unique trait of Geophagini, and its function remains unknown. Because EBL-bearing genera such as *Geophagus*, *Gymnogeophagus*, ‘*Geophagus*’ *steindachneri* and *Satanoperca* are specialized substrate-sifters and also include species that are mouth brooders, the EBL could have evolved in association with either of these behaviors. In this paper we use a previously derived phylogeny [16] and morphological and dietary analyses to: 1) establish associations between diet and morphology in the major lineages of Geophagini and 2) test hypotheses of association of the EBL with benthic feeding [25] and/or mouth brooding [24].

## Results

### Feeding ecology of Geophagini

**Figure 1:**
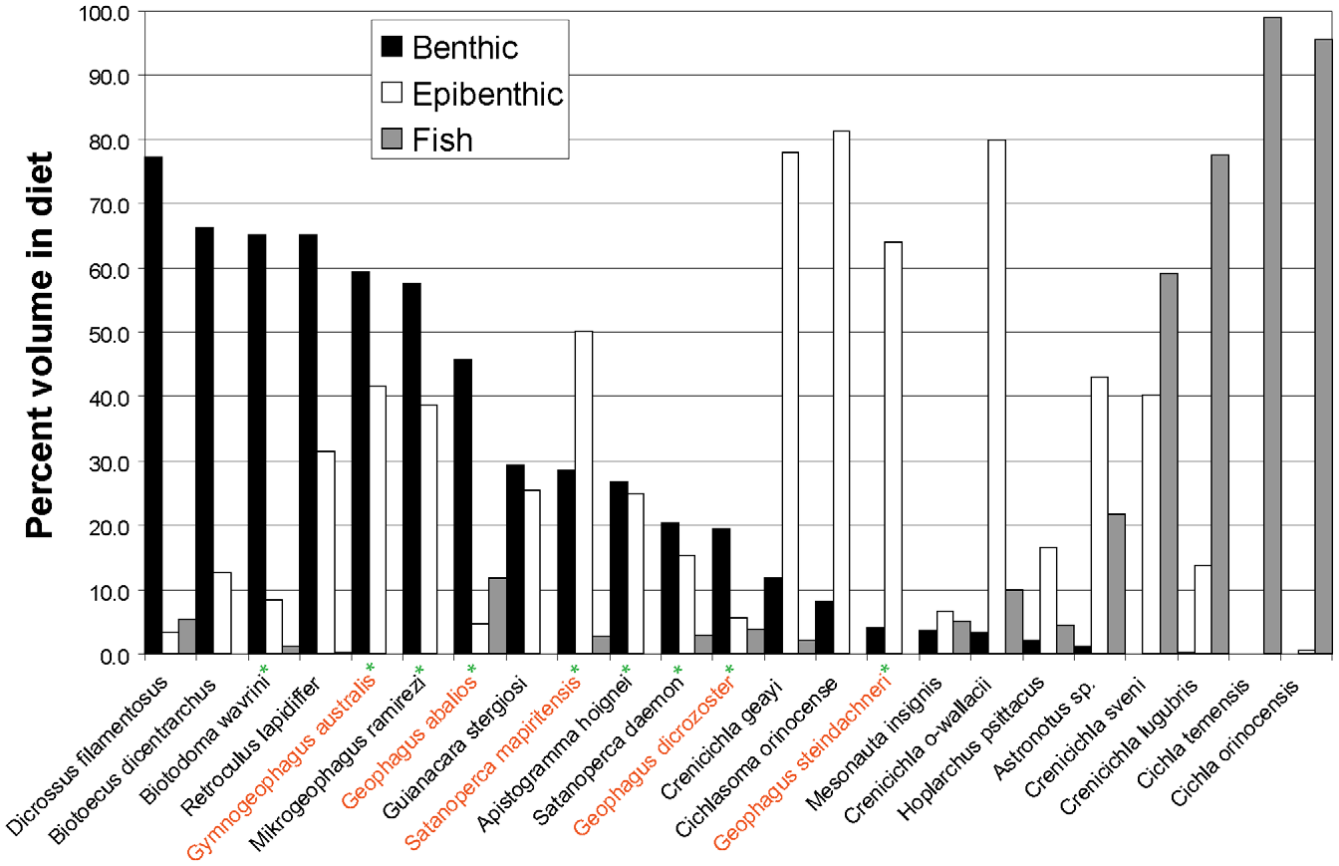
Percent volume for the three dominant diet categories of the 23 species studied. Categories are: benthic prey, epibenthic prey and fish. Species are arranged in decreasing order of percent benthic diet to illustrate the apparent pattern of exclusion between benthic and fish diets. Green stars show the distribution of the EBL, and orange species names indicate mouthbrooding lineages. See text and Table 1 for more details.

Analyses of digestive tract contents revealed that most taxa examined fed primarily on three diet categories, benthic invertebrates, epibenthic invertebrates, and fish, with a comparatively small number of species feeding on the other four dietary categories (Table 1, Fig. 1, and see Methods, File S2). Most of these latter species were non-geophagine omnivores, with *Hoplarchus psittacus* and *Mesonauta insignis* (Heroini) consuming large amounts of detritus, and *Astronotus* sp. consuming a mixture of fish, surface and epibenthic invertebrates. Among geophagines, *Apistogramma hoignei*, *Biotodoma wavrini*, *Guianacara stergiosi* and the two species of *Geophagus sensu stricto* consumed variable amounts of detritus, but their diets still contained large fractions of benthic invertebrates. The greatest fractions of benthic invertebrates (≥20%) were consumed by geophagine species and by the basal genus *Retroculus*, whereas fish consumption was almost exclusively restricted to the two species of *Cichla* and the two large-bodied, predatory geophagines *Crenicichla sveni* and *C.* “sp. Orinoco-lugubris”. Epibenthic prey dominated the diet of the smaller-bodied *Crenicichla geayi* and *C.* “sp. Orinoco-wallacii” as well as those of *‘Geophagus’ steindachneri* and the cichlasomatine *Cichlasoma orinocense*. Nevertheless, epibenthic invertebrates were consumed in fairly large amounts by a large number of taxa, even when their diet was dominated by other food categories.

**Table 1:** Percent volume of diet categories in 23 species of Neotropical cichlids and their phylogenetic signal.

	Diet Category							
	N	Benthic	Epibenthic	Fish	VegetableDetritus	AnimalDetritus	Surface	WaterColumn
TFSI p-values (untransformed branches)		<0.05	0.303	<0.01	0.053	0.313	0.222	0.342
*Dicrossus filamentosus* (Df)	10	77.2	3.2	5.5	0.0	14.1	0.0	0.0
*Biotoecus dicentrarchus* (Bd)	71	66.4	12.7	0.0	10.6	3.2	0.1	0.0
*Biotodoma wavrini* (Bw)	79	65.2	8.4	1.2	12.5	11.4	0.0	0.0
*Retroculus lapidiffer* (Re)	90	65.2	31.6	0.3	1.0	0.8	1.1	0.0
*Gymnogeophagus australis* (Gg)	16	59.4	41.7	0.0	0.0	0.0	0.0	0.0
*Mikrogeophagus ramirezi* (Mk)	28	57.5	38.7	0.0	3.8	0.0	0.0	0.0
*Geophagus abalios* (Ga)	90	45.8	4.7	11.9	20.3	8.4	1.0	1.5
*Guianacara stergiosi* (Gu)	30	29.4	25.4	0.0	15.9	29.3	0.1	0.0
*Satanoperca mapiritensis* (Sm)	30	28.7	50.1	2.8	14.4	2.8	0.0	0.0
*Apistogramma hoignei* (Ah)	242	26.8	25.0	0.0	26.7	9.4	10.0	0.8
*Satanoperca daemon* (Sd)	82	20.5	15.3	2.9	13.7	40.0	0.4	0.0
*Geophagus dicrozoster* (Gd)	65	19.4	5.7	3.8	36.1	27.0	0.5	0.0
*Crenicichla geayi* (Cg)	134	11.9	78.0	2.2	0.6	0.1	1.1	4.2
*Cichlasoma orinocense* (Ch)	300	8.1	81.2	0.0	4.3	0.2	2.5	0.8
*Geophagus steindachneri* (‘G.’s)	2	4.1	64.0	0.0	11.6	20.2	0.0	0.0
*Mesonauta insignis* (Me)	65	3.7	6.7	5.1	58.5	20.0	5.5	0.6
*Crenicichla* sp. ‘Orinoco-wallacii’ (Cw)	148	3.3	80.0	10.0	1.3	3.9	1.5	0.0
*Hoplarchus psittacus* (Ho)	36	2.0	16.6	4.4	24.7	52.3	0.0	0.0
*Astronotus sp.* (As)	99	1.2	43.0	21.9	1.0	4.7	25.3	2.9
*Crenicichla sveni* (Cs)	41	0.0	40.2	59.1	0.0	0.2	0.0	0.5
*Crenicichla* sp. ‘Orinoco- lugubris’ (Cl)	325	0.2	13.8	77.6	0.7	2.4	5.2	0.0
*Cichla orinocensis* (Co)	125	0.0	0.7	95.6	0.0	0.0	0.0	1.4
*Cichla temensis* (Ct)	143	0.1	0.0	99.1	0.0	0.0	0.0	0.0

**Figure 2:**
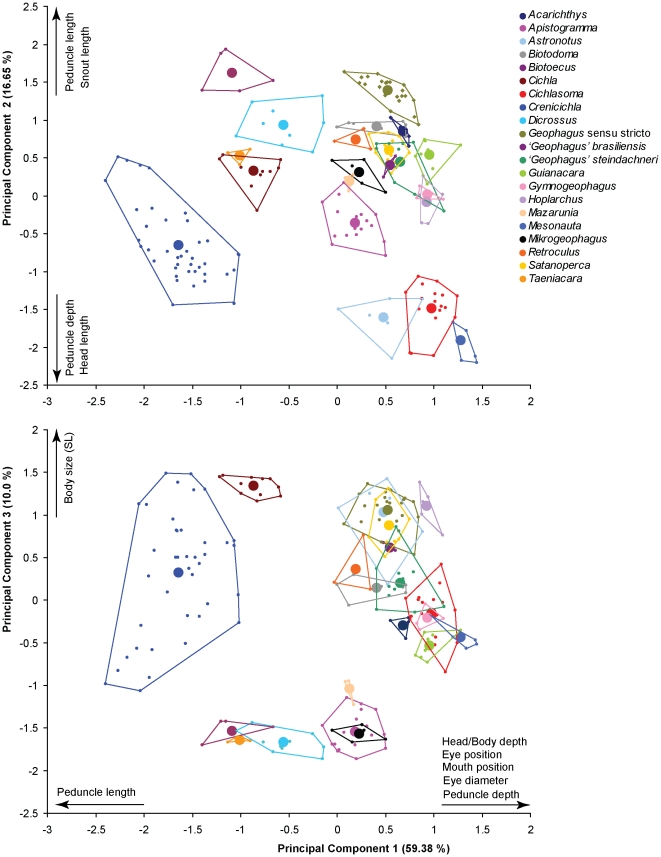
Graphic representation of the first three principal components of morphology. PCA analysis included Ln(SL) and 9 size-corrected morphological variables for 55 species in 21 genera of South American cichlids, including 15 of 17 geophagine genera (missing genera are *Teleocichla* and *Crenicara*). Genera are labeled by color as per the inset legend. Polygons represent the total morphospace for each genus, with small points depicting each individual score and large points their centroid. Scores for morphological variables increase in the direction of the arrows. (See Table S3 for species list, sample sizes, morphological eigenvector values and score values for each individual fish).

Species are arranged in decreasing order of importance of benthic invertebrate prey. Unless otherwise indicated, prey categories are invertebrate prey; N is sample size; see text and Fig. 2 for more details.

In summary, of the seven diet categories originally defined (see Materials and Methods, File S2) three trophic categories (benthic, epibenthic and fish) were most commonly found in the diet of geophagine cichlids examined. When species were compared based on dietary percentages among these three dominant categories, a general pattern was revealed. There was little overlap in species with diets consisting predominantly of benthic prey versus fish, even though both groups consumed significant fractions of epibenthic invertebrates (Fig. 1). In accordance with this pattern of mutual exclusion between benthivory and piscivory, tests of phylogenetic serial independence indicated only these two categories were significantly constrained by phylogeny (Table 1). All species of the Geophagini and Retroculini were benthivorous, and specialized piscivory was restricted to Cichlini and species from the geophagine genus *Crenicichla*.

### Ecomorphology of geophagine cichlids

Morphometric analysis of 10 variables of external morphology for 55 species in 21 genera revealed a strong pattern of morphological divergence among Neotropical cichlid genera (Fig. 2, and see Table S3). Despite some amount of interspecific morphometric variation within genera, this analysis clearly reveals that intergeneric separation greatly exceeds interspecific variation, with each genus separated from all other genera along at least one dimension of multivariate space. For example, even though the geophagine genus *Gymnogeophagus* overlaps with the heroine genus *Hoplarchus* along PC axis 1, the two taxa are clearly separated along PC axis 3. Similarly, small-bodied genera overlap along PC 3, but are clearly separated from each other along PC1 and PC2. Overall, morphological variation within genera is very limited, especially in comparison to variation between genera. Even the most morphologically variable genus, *Crenicichla*, had no overlap with other genera, suggesting substantial ecomorphological divergence from other Neotropical lineages.

**Figure 3:**
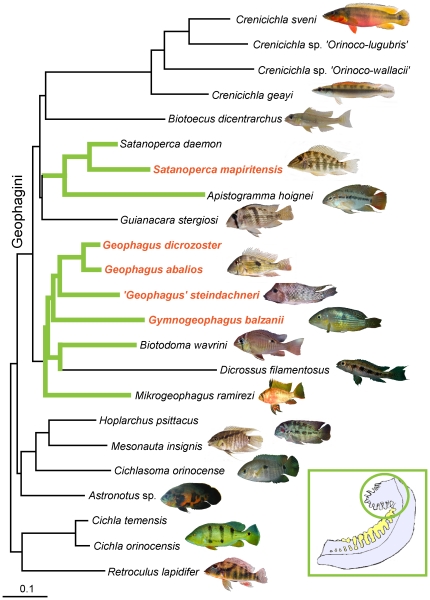
Pruned tree with 23 taxa used for diet and morphology analyses. Photographs depict representatives of all genera analyzed in this study (except *Dicrossus*) and illustrate morphological variation among genera of Neotropical cichlids (see also Fig. 2). Species shown do not necessarily represent the same species studied. Inset is a diagrammatic representation of the first gill arch with the geophagine epibranchial lobe (EBL) highlighted within the circle. Green branches show the distribution of the EBL, and orange species names indicate mouthbrooding lineages. Photographs by H López-Fernández, K. M. Alofs and A. Lamboj.

The remainder of our analyses tested relationships between morphology and diet in the 23 taxa included in our pruned phylogeny (Fig. 3, Table S1). Procrustes superimposition of phylogenetically uncorrected matrices of diet and morphology showed a significant association between the two data types (m_12_ = 0.801, *p*<0.03), revealing a strong ecomorphological correspondence. The same analysis, using independent contrasts of the same data to account for phylogenetic relatedness, was not significant (m_12_ = 0.9572, *p* = 0.94, not shown).

**Figure 4:**
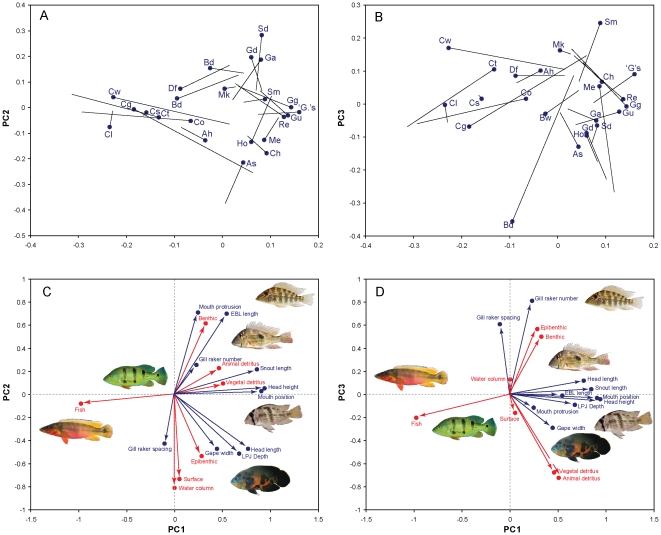
Procrustean superimposition of three first principal components of morphology and diet. PROTEST permutation [62] revealed a significant association between diet and morphology without phylogenetic correction (m_12_ = 0.8015, *p*<0.03). The analysis was not significant after phylogenetic correction (m_12_ = 0.9572, *p* = 0.94, not shown). **a** and **b**, Procrustes superimposition plots of morphological variables (blue dots) and diet categories (end point of solid lines) for PC1 v. PC2 and PC1 v. PC3 scores, respectively. Solid lines represent residuals after the Procrustes superimposition procedure. Species codes correspond to the two-letter codes in Tables 1 and 2. **c** and **d**, directions of variation of morphological variables (blue) and diet categories (red) as indicated by their eigenvectors in the PCA analysis. The original diet eigenvectors have been transformed using the rotational matrix from the Procrustes procedure to maximize the association with the morphological eigenvectors. Photographs depict genera representing the associations between diet and morphology in their region of combined multivariate space. Photographs by H. López-Fernández, K. M. Alofs and A. Lamboj.

Collectively, the first three principal components from the PCA without phylogenetic correction (Table 2, Fig. 4 and see Table S1) explained 72.8% and 74.4% of the variance in morphology and diet, respectively. Phylogeny-corrected analyses explained only 59.7 and 66.8 percent of the variance along the first three PC axes of morphology and diet, respectively (Table S2). Procrustes superimposition, as revealed by the magnitude of the residual displacement of the diet on morphology matrices, shows that while there is an overall tight association between the two types of variables, there is also a large amount of variance in diet that remains unexplained by morphological variables (Fig. 4a,b). Nevertheless, the combination of morphological and rotated diet vectors in the PCA (Table 3) reveal clear patterns of association between morphological traits and diet categories (Fig. 4c,d). Principal component 1 partitioned ecomorphological variation into a gradient bracketed by two phenotypes: 1) fishes with relatively long and shallow heads, relatively thin lower pharyngeal jaws (LPJs) and short snouts, with the mouth being terminally to dorsally positioned and feeding strongly on fish (*Crenicichla*, *Cichla*); and 2) fishes with short and deep heads, thick LPJs, long snouts and ventrally oriented mouths (e.g. *Guianacara*, *Gymnogeophagus*) that feed mostly on benthic and epibenthic prey and detritus (Fig. 4c,d). However, examination of the Procrustes residuals also indicates that, in some cases, taxa with morphologies tied to certain types of diet are actually feeding on a different category of prey. This is especially clear in the case of *Crenicichla geayi* and *C.* “Orinoco-wallacii”, relatively small species with a piscivorous morphology that feed heavily on epibenthic invertebrate prey (Fig. 2, and note the origin and magnitude of the diet residuals in Fig. 4a,b). In the case of *Cichla*, analysis of the residuals indicates that these strictly piscivorous species have a less specialized morphology than piscivorous *Crenicichla* “Orinoco-lugubris” and *C. sveni* that reveal a better fit between diet and morphology.

**Table 2:** Principal components analysis eigenvectors for 7 diet categories and 10 morphometric variables associated with feeding.

	Morphological variables PCA eigenvectors					
	Uncorrected PCA			Phylogeny-corrected PCA		
	PC1	PC2	PC3	PC1	PC2	PC3
Eigenvalue	4.117	2.005	1.158	2.885	1.904	1.179
Cumulative percent variance explained	41.2	61.2	72.8	28.8	47.9	59.68
Head Length	0.768	−0.474	0.120	0.337	00.690	−00.244
Head height	0.939	0.052	−0.043	0.706	−0.262	−0.121
Gape Width	0.444	−0.472	−0.291	−0.216	0.579	0.060
Snout Length	0.862	0.217	0.092	0.732	0.404	0.216
Mouth Position	0.908	0.028	−0.033	0.742	−0.123	0.131
No. Gill rakers in 1^st^ Ceratobranchial	0.231	0.255	0.810	0.330	0.126	−0.698
Gill raker spacing in 1^st^ Ceratobranchial	−0.104	−0.428	0.608	−0.484	−0.901	0.535
Length of the Epibranchial lobe	0.545	0.701	−0.011	0.670	0.162	0.353
Depth of lower pharyngeal jaw	0.676	−0.515	−0.089	0.393	0.660	0.377
Mouth protrusibility	0.247	0.708	−0.116	−0.438	0.722	−0.402

	Diet categories PCA eigenvectors					
	Uncorrected PCA			Phylogeny-corrected PCA		
	PC1	PC2	PC3	PC1	PC2	PC3
Eigenvalue	2.184	1.572	1.449	1.990	1.426	1.256
Cumulative percent variance explained	31.2	53.7	74.4	28.43	48.81	66.75
Benthic	0.404	0.656	−0.375	−0.365	−0.803	0.516
Vegetable detritus	0.627	−0.629	−0.060	0.779	0.001	0.499
Animal detritus	0.696	−0.515	−0.007	0.837	−0.185	0.225
Epibenthic	−0.532	0.036	−0.637	−0.109	−0.577	0.550
Fish	−0.421	0.058	0.904	−0.656	0.0135	0.460
Surface prey	−0.470	−0.571	−0.137	0.260	0.786	0.190
Water column prey	−0.680	−0.386	−0.251	−0.203	0.659	0.374

PCA results are given for analyses with and without phylogenetic correction. See text for details.

**Table 3:** Rotational matrix and rotated diet PCA eigenvectors from Procrustes superimposition of diet and morphology.

	Procrustes rotational matrix (H)		
	−0.459	−0.770	0.443
	0.268	−0.595	−0.758
	0.847	−0.229	0.480

	Procrustes rotated eigenvectors		
Diet category	PC1	PC2	PC3
Benthic	0.327	0.615	0.499
Vegetable detritus	0.507	0.095	−0.725
Animal detritus	0.463	0.228	−0.695
Epibenthic	0.285	−0.534	0.568
Fish	−0.975	−0.082	−0.203
Surface prey	0.053	−0.734	−0.159
Water column prey	0.004	−0.811	0.129

Principal component 2 also revealed associations between distinctive phenotypes and diets: 1) fishes with thick LPJ bones, relatively long heads, wide gapes with low mouth protrusion and widely spaced gill rakers feeding on epibenthic, surface or water column prey (e.g. *Astronotus*, *Cichlasoma*), and 2) exclusively geophagine taxa with relatively shorter heads and narrower gapes, highly protrusible mouths, thin LPJ plates and epibranchial lobes (*Satanoperca*, *Geophagus*) that specialize on benthic invertebrates (Table 2, Fig. 4c). Thus PC2 separates benthic from epibenthic feeders, even when they had similar scores along axis 1, and suggests that different morphologies are involved in the consumption of these two types of food. Finally, morphology and diet associations along PC 3 revealed some association between numerous, widely separated gill rakers and benthic or epibenthic diets, as well as a correlation between mouth protrusion and wide gapes and consumption of detritus. Nonetheless, the magnitude of the Procrustes residuals (Fig. 4b) suggests that diet-morphology associations along this last axis are not particularly tight.

Seven of the ten retained morphological variables revealed significant phylogenetic signal as determined by TFSI tests (Table 4). Several variables with strong associations with benthic feeding (e.g. EBL, mouth position) or piscivory (e.g. head length, mouth position) were significantly correlated with phylogeny (Table 4). Mouth protrusion, gape width, and gill raker number, each associated with more than one type of prey, were not phylogenetically constrained, suggesting that these morphological traits facilitate consumption of a variety of different prey and are not necessarily associated with trophic specializations restricted to particular clades.

**Table 4:** Mean values and phylogenetic signal of the 10 untransformed morphometric variables used in comparative analyses.

	Morphometric variables												
	N	SL	SL Range	Head length	Head height	Gape width	Snout length	Mouth protrusion	Mouth position (°)	Mean No. Gill rakers	Gill raker space	Epibranchial lobe length	LPJ Depth
TFSI p-values (untransformed branches)				<0.05	<0.01	0.32	<0.01	0.06	<0.01	0.44	<0.05	<0.01	<0.01
*Apistogramma hoignei* (Ah)	5	28.8	27.3–33.4	10.1	6.3	1.5	3.8	0.8	49.3	1.4	0.16	1.02	0.68
*Biotoecus dicentrarchus* (Bd)	5	32.4	28.4–35.9	9.6	5.7	1.6	4.3	1.5	37.8	0.0	0.00	0.93	0.42
*Dicrossus filamentosus* (Df)	5	30.3	27.2–34.6	9.1	5.1	1.0	3.4	1.8	49.0	4.0	0.28	-*	0.37
*Mikrogeophagus ramirezi* (Mk)	5	31.2	28.7–33.8	10.0	7.5	1.2	4.2	1.6	53.2	7.0	0.26	0.84	0.47
*Crenicichla geayi* (Cg)	5	114.9	92.8–136.0	35.9	13.7	10.4	14.3	3.1	25.3	8.6	0.94	-	2.06
*Crenicichla sveni* (Cs)	5	136.4	104.8–193.0	45.3	19.3	13.2	20.0	3.5	26.2	8.8	1.67	-	1.85
*Crenicichla* ‘Orinoco-wallacii’ (Cw)	5	48.9	43.0–55.4	14.7	5.2	2.2	6.3	0.9	24.6	8.8	0.37	-	0.58
*Crenicichla* ‘Orinoco-lugubris’ (Cl)	5	213.0	187.0–244.0	64.0	27.1	19.1	30.8	2.0	24.6	10.0	2.07	-	3.01
*Geophagus abalios* (Ga)	5	170.6	144.0–211.0	54.8	57.0	14.7	33.6	5.3	55.6	12.0	1.38	9.38	3.39
*Geophagus dicrozoster* (Gd)	5	178.0	164.0–205.0	55.5	55.2	15.1	35.2	4.7	53.8	12.0	1.17	10.28	3.53
*Satanoperca daemon* (Sd)	5	157.6	119.0–190.0	54.4	45.7	12.5	35.8	5.2	43.0	18.0	0.55	9.92	2.72
*Satanoperca mapiritensis* (Sm)	5	147.6	137.0–164.0	50.6	46.5	13.0	33.1	3.5	47.8	15.4	2.97	8.18	3.00
*Biotodoma wavrini* (Bw)	5	96.5	84.0–104.5	29.0	29.8	5.9	16.3	2.5	52.6	6.8	0.78	2.92	1.71
*Guianacara stergiosi* (Gu)	5	65.4	59.6–69.0	22.6	24.2	5.2	13.6	2.6	58.0	6.8	0.57	-	1.94
*Astronotus* sp. (As)	5	203.2	184.0–228.0	72.5	55.4	23.8	30.6	3.0	47.8	9.8	1.71	-	5.82
*Hoplarchus psittacus* (Ho)	5	185.4	148.0–217.0	66.3	65.5	16.5	36.8	2.0	56.2	8.6	1.29	-	4.17
*Cichla orinocensis* (Co)	2	205.0	199.0–211.0	65.7	42.0	18.4	30.7	3.6	38.5	17.0	1.72	-	4.66
*Cichla temensis* (Ct)	3	223.3	206.0–237.0	71.3	39.1	15.3	34.4	2.9	32.7	16.0	2.13	-	4.57
*Cichlasoma orinocense* (Ch)	5	79.8	62.2–99.2	30.0	25.3	7.0	13.3	2.0	56.0	7.4	1.22	-	2.32
*Mesonauta egregius* (Me)	5	65.3	61.8–71.4	24.4	21.9	4.7	13.8	1.4	51.0	5.2	0.84	-	1.55
*Geophagus' steindachneri* (‘G.’s)	2	80.9	79.7–82.1	31.8	25.8	7.0	16.2	2.2	52.0	14.0	0.67	4.42	2.21
*Gymnogeophagus balzanii* (Gg)	4	78.7	65.5–98.3	28.5	29.4	8.1	16.5	2.1	53.3	8.6	0.59	3.43	1.64
*Retroculus lapidifer* (Re)	1	141.0	-	51.7	36.6	11.8	31.2	2.3	69.0	12.0	1.14	4.00	3.80

All values are in millimeters except where indicated. See text for more details.

* Species identified with a “-” in this column lack an epibranchial lobe.

### Association of the EBL with mouth-brooding and patterns of diversification

When Pagel's [27] test of association between EBL and mouth brooding was performed on the 157 taxa from [16] (−Ln uncorrelated = −34.59, −Ln correlated = −28.36, difference = 6.23, p<0.01) and 635-taxa topology (−Ln uncorrelated = 62.53, −Ln correlated = 58.27, difference = 4.26, p<0.01), the likelihood difference between the uncorrelated and correlated models of evolution was significant. A significant association was lost by a small margin in the 350-taxa case (−Ln uncorrelated = 39.58, −Ln correlated = 35.43, difference = 4.15, p = 0.065). Overall, these results suggest that the presence of the EBL in geophagine cichlids is indeed associated with oral incubation of the eggs and/or fry. Nevertheless, the sister diversification test indicated that clades of Geophagini with an epibranchial lobe were no more diverse than clades without the lobe (Binomial sign-test p = 0.75 for both the 350 and 635-taxa topologies).

## Discussion

### Diet-morphology correlations in Geophagini

Procrustes superimposition analysis of diet and morphology datasets revealed clear associations between specific morphological attributes and particular diet categories. Most notably, we found a tight correspondence between the specialized benthivorous diet of certain geophagine genera (e.g. *Geophagus*, *Satanoperca*) and the presence of the epibranchial lobe. Diets dominated by benthic organisms and fish, as well as several associated morphological attributes were phylogenetically constrained as indicated by the TFSI tests (Tables 1 and 4). These phylogeny-dependent correlations suggest that at least some ecomorphological attributes are functionally linked within certain clades. Use of independent contrasts, a method that corrects for statistical non-independence due to phylogeny [28], eliminated significant diet-morphology correlations found in the uncorrected data. Loss of statistical significance in the phylogeny-corrected analysis could be due to reduced degrees of freedom resulting from the fact that phylogeny-dependent correlations are limited to very few clades. For instance, the EBL is present in only two clades of Geophagini with very similar ecomorphological associations, statistically equating these associations to a single evolutionary event. Statistically, this limits our ability to strongly establish correlations between the EBL and its possible ecological functions. Moreover, the strong phylogenetic effect suggests that, in the case of benthivory and piscivory, ecomorphologically similar taxa within clades share a common evolutionary history, supporting the idea that the correlations we found reveal ancestral ecomorphological patterns. This is consistent with the finding that morphological variation in Neotropical cichlids appears to be much greater among genera than within genera (Fig. 2).

This interpretation is consistent with paleontological evidence in the form of a geophagine fossil (†*Gymnogeophagus eocenicus*), dating to the Eocene, that is nearly indistinguishable from modern species in the genus [29]. Thus both our results and fossil evidence indicate morphological and ecological differentiation at high phylogenetic levels followed by stasis at lower levels. This pattern suggests a potential decoupling between mechanisms generating higher level diversity in deep time and more recent or ongoing processes responsible for species-level diversification. High phenotypic differentiation near the base of the tree (i.e. at the genus level) is compatible with ancient diversification in the presence of ecological opportunity. Presumably, basal lineages differentiated to exploit particular niches and thus resulted in specialized ecomorphological combinations [17], [30], [31]. Eventually, morphological stasis ensued because the ability of newly emerged lineages to persist over time diminished along with the number of available niches [31]–[33]. This scenario is consistent with historical-biogeographical reconstructions of the evolution of other Neotropical freshwater fishes as well. Evidence shows that many fossil Neotropical fishes of considerable age are essentially identical to modern forms and presumably have been part of similarly-assembled communities since at least the Paleogene [2], [7], [34].

### Ecomorphological associations in geophagine cichlids

We found a clear association between morphology and substrate sifting, and among geophagines this behavior is associated with 1) short, deep heads, 2) ventrally oriented, highly protrusible mouths, and 3) modifications of the pharyngeal apparatus, including weak pharyngeal jaws and presence of the epibranchial lobe (Fig. 3). In combination, the ecomorphological attributes of substrate-sifting geophagines point towards specialized benthic-feeding behavior. The only other feeding group that showed a clear ecomorphological pattern was the piscivores represented by the basal genus *Cichla* and the geophagine genus *Crenicichla*. Piscivores tended to occupy a distinct region of morphospace characterized by elongated and shallow heads with only marginally protrusible mouths.

Although our study is purely correlative, some of these axes of ecomorphological variation have obvious functional interpretations derived from biomechanical studies. Piscivorous cichlids have an orobranchial morphology known to be associated with efficient ram feeding [35]. The positions of species along PC1 of our morphological analysis coincides remarkably well with the functional morphology that Wainwright et al [35] identified along a ram-distance axis based on performance of cichlid taxa in feeding trials. Along PC2, geophagine substrate sifters have traits that are traditionally associated with suction feeding, such as a narrow mouth [36], [37], as well as stronger biting force associated with short, high head [38], and post-capture handling of prey through winnowing, which involves both oral jaw protrusion and pharyngeal manipulation by sieving of prey-substrate mix through the gill rakers and possibly the EBL [19]. Jaw protrusion in piscivores is thought to increase the efficiency of fast ram-attacks on elusive prey [35], [39], yet it was clearly associated with benthic feeding in our analyses, suggesting that protrusion is used in different ways by piscivores and benthivores. Subterminal jaw protrusion in benthic feeders such as Geophagini is clearly important for capture of individual food items and not just for winnowing, as pointed out by Hulsey and García de León [39] for the Central American Heroini cichlid genera *Thorichthys* and *Astatheros*. *Retroculus lapidifer* had one of the highest proportions of benthic prey in its diet, and this species grouped with several geophagines in multivariate space (Fig. 2, Table S1). Moreover, *Retroculus* has pharyngeal modifications of the first epibranchial that are analogous to the geophagine EBL [15]. Substrate sifting also is observed in distantly related African cichlids like *Chromidotilapia* [40] that have lobe-like modifications of their pharynx, albeit in the second epibranchial instead of the first one as in Geophagini and Retroculini. These convergences in feeding behavior and morphology provide additional support for the idea that the geophagine EBL facilitates ingestion of benthic invertebrates.

In theory, ecomorphological specialization limits an organism's ability to exploit alternative resources. This basic tradeoff was supported in a recent biomechanical study of centrarchid piscivores of the genus *Micropterus* [41]. We speculate that species of the genus *Cichla* and possibly some large predatory species of *Crenicichla* (See Figs. 2, 3, 4) may be limited in their trophic flexibility by a specialized morphology. Whether or not such restrictive specialization may also be present in substrate sifters remains unclear. In our dataset, specialized benthic feeders (i.e. consuming both benthic and epibenthic prey) only occasionally consumed significant amounts of other prey types (Fig. 1 and Table 1). The only example of an ecomorphologically variable clade within the Geophagini is the genus *Crenicichla*. As our genus-level comparisons illustrated (Fig. 2), *Crenicichla* shows a remarkable degree of interspecific diversification when compared to other geophagine genera. Among the four species of *Crenicichla* we studied, the two largest ones were predominantly piscivorous and the two smallest species had diets dominated by epibenthic prey despite their largely piscivorous morphology (Fig. 3, and see Fig. 4a,b). With more than 100 species, the genus *Crenicichla* apparently evolved into a region of morphospace that either allowed, or was a precursor for, greater ecological diversification when compared with related clades.

Although several morphological attributes in our dataset were found to be phylogenetically constrained, the most remarkable is the epibranchial lobe. This structure is present in several genera within two major clades of Geophagini, apistogrammines and geophagines, suggesting the EBL appeared early in the evolution of the group. We found the EBL is associated with feeding as shown by multivariate analyses of ecomorphological data, and it also is associated with mouth brooding as shown by Pagel's character correlation test, suggesting that the lobe may be an adaptation associated with either behavior. Despite these correlations, the EBL does not appear to be associated with increased lineage diversification in geophagines or any of its subclades.

No functional morphological model is available to explain how the geophagine epibranchial lobe may be involved in either mouthbrooding or substrate sifting. Because several species of non-geophagines that lack epibranchial lobes (e.g. the cichlasomatine genus *Bujurquina*) are mouth brooders, the functional significance of the EBL for mouth brooding is uncertain. Mouth brooding is widespread among cichlids, and it is especially common among African clades with modifications of pharyngeal features that are non-homologous with those of geophagines. A confounding factor is that many cichlids that are mouth brooders are also benthic feeders, thus making it difficult to determine whether the EBL or analogous structures are correlated with one or both of these behaviors. To our knowledge, no study has addressed the functional role of these pharyngeal modifications for either feeding or mouth brooding. At this point, we prefer to treat the correlation between the EBL and benthic feeding or mouth brooding as interesting patterns that need further study, without dismissing the possibility that this structure might have different or multiple functions depending on the species. The data analyzed in the present study cannot discern the order in which these associations might have evolved. Comparative analysis of the biomechanics of benthic-feeding and mouthbrooding in geophagines, with a particular focus on the role of the EBL, should prove a fruitful avenue for future research.

## Materials and Methods

### Ethics statement

This work was based on specimens available at various Natural History Museums and were collected well before this study. When collection of specimens was performed specifically for this work, fish were collected under the following Animal Use Protocols (AUPs):

1. AUP 2005-117, “Adaptive radiation and evolutionary convergence in Neotropical Cichlids” to Kirk O. Winemiller; approved by the University Laboratory Animal Care Committee, Texas A&M University and valid from 5/23/2005 to 5/22/2008
2. AUP 2008-60, “Fish assemblage structure and functional trait diversity along a longitudinal fluvial gradient” to Kirk O. Winemiller; approved by the Division of Research and Graduate Studies – Office of Research Compliance, Texas A&M University and valid from 4/29/2008 to 4/28/2011
3. AUP 2011-02. “Comparative evolutionary ecology of Neotropical cichlid fishes” to Hernán López-Fernández; approved by the Animal Care Committee, Royal Ontario Museum and valid from 4/29/2011 to 4/29/2012

### Phylogenies and taxonomic data used for comparative analyses

All analyses were based on the multi-locus, genus-level phylogeny of Neotropical cichlids presented by López-Fernández et al [16]. Although other hypotheses of geophagine relationships are available [13], we chose the tree by López-Fernández et al [16] because it has a larger species-level sample size and branch-lengths based on likelihood methods, which are necessary in quantitative comparative methods (see below). The phylogeny by López-Fernández et al [16] includes virtually all major lineages of Neotropical cichlids, including all described genera in the seven tribes of the Neotropical subfamily Cichlinae. Readers are referred to that paper for details on the topology, the methods used for its construction and its derived taxonomic nomenclature. Comparative analyses of associations between diet and morphology were based on a pruned version of the tree that included 23 taxa for which we were able to collect detailed morphometric and dietary data (see below). This pruned tree (Fig. 1, File S3) comprises 11 of the 17 putative geophagine genera, including all major clades in the tribe Geophagini as well as representatives of Cichlini, Retroculini, Astronotini, Cichlasomatini and Heroini for comparison.

Beyond direct correlations between diet and morphology, testing hypotheses of associations of mouthbrooding with the EBL required a broader phylogenetic framework than represented in either López-Fernández et al.'s [16] phylogeny or our pruned ecomorphological topology (see above). Because there is no available species-level phylogeny for either Geophagini or Cichlinae, we used López-Fernández et al.'s phylogeny as a “backbone” of genus-level relationships and combined it with a list of estimated total species of both Geophagini and all Neotropical cichlids. We included every species in each genus by creating a polytomy at the species level, obtaining a tree with the complete tip diversity for each clade. Although this method has the obvious limitations of being both uninformative at the species level and lacking terminal branch lengths for these species, it still provides an approximation to the distribution of diversity within the Neotropical cichlid phylogeny. Similar approaches that include diversity at the tips, while assuming no knowledge of species-level relationships, have been implemented in similar types of analyses [42], [43]. Using this approach, we constructed a species-level tree of Geophagini (350 species, File S5) that also included representatives of the tribes Cichlini (15 species), Retroculini (4 species) and Chaetobranchini (4 species), which were used as outgroups. In addition, we created a tree with 635 terminals (File S5) that included all species of Neotropical cichlids. Generic assignments in this tree are based on López-Fernández et al. [16]. Both trees were rooted with the Retroculini-Cichlini clade. Trees obtained with this method allowed for mapping the presence or absence of the EBL and mouthbrooding among clades within Geophagini and Neotropical cichlids in general.

A taxonomic list initially obtained from FishBase [44] that included only validly described taxa was used to create the species polytomies. There is, however, ample evidence that many Neotropical species of cichlids remain undescribed [8], [16], [45]. We attempted to address this issue by including additional taxa that are known to be undescribed species. Whenever possible, unnamed putative species were added following information from museum collections in which undescribed species have been identified, particularly at the Museo de Ciencias Naturales de Guanare (MCNG, Venezuela, D.C. Taphorn, pers. Comm.) and the Royal Ontario Museum (ROM, Canada, HLF pers. obs.). Additionally, some catalogues were used to identify species that are available in the aquarium trade for which both photographs and reasonably reliable localities of origin are available [46]–[49]. In all cases, putative undescribed species were added only if examination of museum specimens or photographs left no doubt of the distinctness of each taxon. This practice, combined with the inaccessible nature, extremely high diversity, and relatively scarce field exploration of many Neotropical areas, especially in South America, more than likely resulted in an underestimation of the actual Neotropical cichlid diversity, but it is impossible at the moment to obtain a reliable figure only from the scientific literature. For the purposes of our tests, an underestimation of species diversity should not alter the general trends observed in our analyses.

### Ecomorphology of feeding in geophagine cichlids

We performed stomach contents and morphometric analyses on wild-caught cichlid specimens stored in the ichthyology collections of the Museo de Ciencias Naturales de Guanare (MCNG, Universidad de Los Llanos, Venezuela), the Royal Ontario Museum (ROM, Canada), and the Texas Cooperative Wildlife Collection (TCWC, Texas A&M University, USA). To reduce the confounding effects of intraspecific allometry and ontogenetic diet changes, only adult specimens were included in the analyses. In a few cases, we could not obtain ecological data for the same species included in the phylogeny. However, because our analyses focus on genus-level ecomorphological differentiation, in these cases we used individuals of different species than those in the phylogeny to describe the ecology and morphology of the genus (and see Results, Fig. 2).

Diet composition was quantified by volume based on food items found in the anterior half of the digestive tract. Gut contents were separated into the highest-resolution identifiable taxonomic category, and the total fraction of each category was blotted dry and its volume calculated by water displacement following Winemiller [50]. We analyzed 2251 digestive tracts from 21 species (Table 1) and added data from the literature for 90 stomachs of *Retroculus lapidifer* [50]–[53] and 16 of *Gymnogeophagus australis* [54]. A total of 66 identifiable diet items were found in the gut contents and grouped into seven trophic categories: benthic invertebrates, epibenthic invertebrates, vegetative detritus, animal detritus, water-column prey, surface invertebrates, and fish (File S2). These categories are sufficiently distinct that they should reflect differences in the nutritional value, microhabitats, morphology, size and behavior of prey, as well as consumer foraging strategies.

Morphometric measurements were obtained from one to five individuals of each species in the pruned tree for 23 morphological variables of the head, mouth and pharynx (Files S1 and S4) based on their known or suspected association with feeding [55]. Standard Length (SL) was included as a measure of body size. Morphometric variables were log-transformed as ln(x+1) to increase normality and to account for missing variables in some of the taxa, most notably the absence of the epibranchial lobe in some species. All morphometric variables were size-corrected by regressing the log-transformed values against ln(SL) to remove the effect of body size and to retain components describing body shape [56]. All morphometric analyses were performed on adult specimens to avoid potential confounding effects from allometric changes during ontogeny.

Our ultimate goal of correlating morphology and diet rests on the assumption that most ecomorphological variation in Neotropical cichlids, and particularly geophagines, is observed between genera, whereas intrageneric variation is comparably limited (see above). To test this assumption, we created an expanded morphometric dataset for 207 adult individuals from 55 species in 21 genera of Neotropical cichlids (File S6), including 15 of 17 geophagine genera and all non-geophagine taxa in the pruned tree (see Figs. 1 and 2 and File S1). We used a subset of 10 variables of external morphology corrected for size effects in the same way described for the comparative dataset. We plotted the individual scores and the centroid for each genus on the first three axes of a Principal Components Analysis of the size-corrected external morphology variables plus ln(SL) to account for differences in body size (Fig. 2). If individual scores and/or centroids for each genus were found to occupy different volumes of morphological space, our assumption that interspecific variation within genera does not exceed variation between genera would be supported, and thus conclusions about diet-morphology correlations at the genus-level should be meaningful.

### Phylogenetic independence and comparative analyses of diet and morphology

The presence of significant phylogenetic signal in variables of diet and morphology was assessed using the test for phylogenetic serial independence (TFSI) [57], [58], as implemented in the program Phylogenetic Independence v. 2.0 (http://biology.mcgill.ca/faculty/abouheif/). Results from this test were interpreted as an indication of clade-specific diet or morphological specialization. We also used this test as a gauge to determine whether removal of phylogenetic signal from the continuous variables through phylogenetically independent contrasts (ICs) [36], [59] was effective. Independent contrasts were calculated on percent volumes of diet items and on size-corrected residuals of morphometric variables. We calculated ICs following procedural recommendations by Garland et al. [59], as implemented in the PDAP module for Mesquite [60]. We used untransformed, squared, or log-transformed branch lengths to minimize the correlation between the absolute value of each IC and its standard deviation. Whenever TFSI tests of IC values revealed phylogenetic non-independence, we repeated the IC calculation with a different branch length transformation to ensure that ICs fulfill the method's assumption of Brownian motion [28].

We used the correlation matrix among the initially measured 23 morphological variables (File S4) and the eigenvectors of each variable in a Principal Components Analysis (PCA) as criteria to identify a subset of 10 variables (Table 2) with lowest correlations to each other, and to identify variables with highest PCA eigenvectors as the most robust set of shape discriminators. Ln(SL) was included in these analyses as a way to incorporate body size as an ecomorphological variable complementary to the shape variables represented by the residuals from the SL regressions.

To test the overall fit between the diet and morphology data, we used a Procrustean superimposition approach. The procedure rotates a response matrix (in our case diet) such that concordance with a reference matrix (morphology) is maximized [61]; subsequently, a permutation test (PROTEST) [62] is performed to determine whether the correlation between the two original matrices is different from random. We conducted separate PCA analyses on the set of ten morphological variables and on the seven diet categories, and then built matrices of diet and morphology containing the scores of the first three PCA axes on a correlation matrix of each dataset as a way to reduce “noise” in the data [62]. Significance of the Procrustean superimposition of the diet PCA matrix on the morphology PCA matrix was estimated with 10,000 permutations in PROTEST. The derived rotational matrix (H) was then used to correspondingly rotate the eigenvectors of each diet category in the original PCA matrix so that they could be directly compared with morphological eigenvectors (Table 3). Scores and eigenvectors of both sets of variables were then jointly represented in a Procrustean superimposition plot (Fig. 4). Procrustean superimposition was performed on diet and morphology matrices with and without phylogenetic correction with ICs.

### EBL association with mouth-brooding and patterns of divergence

We tested Haseman's [24] hypothesis that the geophagine epibranchial lobe is associated with mouth brooding. Presence/absence of the EBL and mouth brooding were scored as binary characters for all taxa in the original phylogeny from López-Fernández et al [16] and on both the 350 and 635 taxa trees (File S5). Data for scoring the EBL character states were taken from López-Fernández et al [15], wherein the EBL was found to occur exclusively in the geophagine and apistogrammine clades of Geophagini sensu López-Fernández et al. [16]. The presence of non-homologous modifications of the second epibranchial in *Biotoecus* [15], [63] and the first epibranchial in *Retroculus* [15] was scored as absence of an EBL. Data on mouth-brooding of different taxa were gathered from the scientific literature [64]–[69], field observations by the authors, and aquarium literature [49], with the latter sometimes providing the only information available on the reproductive mode of many cichlids. We used the two binary datasets to perform a modified version of Pagel's [27] test of correlated evolution between binary characters as implemented in Mesquite v. 2.72 [69]. The significance of the difference in likelihood scores between a 4-parameter model of independent character evolution and an 8-parameter model of correlated character evolution was evaluated using a likelihood ratio test based on a null distribution generated with 250 Monte Carlo simulations for the 635-taxon dataset and 1000 for the other two. Because Pagel's test of binary character correlation does not allow missing data, in some cases where monophyly was well supported, a particular behavior was extrapolated to an entire genus from information on one or a few species. We believe this coarse-scale behavioral classification should not profoundly affect the conclusions, but recognize that it does leave space for future analyses that include more detailed descriptions of behavioral traits. Finally, to test whether the presence of the geophagine epibranchial lobe was associated with increased species richness, we used a Binomial sister diversification test that compares species richness in clades with and without an EBL, as implemented in Mesquite [70].

## Supporting Information

Table S1 Species scores in a Principal Components Analysis and Procrustes Superimposition of 7 diet categories and 10 morphometric variables associated with feeding without phylogenetic correction. See text for explanation of the methods and Table 3 for rotation matrix and Procrustes rotation matrices for diet and morphology eigenvector values.
(DOC)

Table S2 Species scores in a Principal Components Analysis of 7 diet categories and 10 morphometric variables associated with feeding with phylogenetic correction. Results are not given in the main text because correlations between diet and morphology disappear after phylogenetic correction. See text for details.
(DOC)

Table S3 PCA Eigenvectors and individual scores for a dataset of 10 morphological variables for 207 individuals in 21 genera and 55 species of South American cichlids, including 15 geophagine genera. See text and Figure 2 for details. Institutional abbreviations are as follows: ANSP - Academy of Natural Sciences of Philadelphia, USA; FMNH: Field Museum of Natural History, USA; INPA: Fish collection of the Instituto Nacional de Pesquisas da Amazonia, Brazil; MCNG: Museo de Ciencias Naturales de Guanare, Venezuela; MCP: Museu de Zoologia do Universidad Pontificia do Rio Grande do Sul, Brazil; ROM: Royal Ontario Museum, Canada; UWI: Zoology collection of the University of the West Indies, Trinidad and Tobago.
(DOC)

File S1 Description of morphometric measurements taken in this study. The fist list includes the intial 23 variables measured in the comparative study of morphology and diet correlations. The second list shows the measures used in the comparison of intrageneric versus intergeneric morphometric variation illustrated in Fig. 2.
(DOC)

File S2 Prey items included in each diet category.
(DOC)

File S3 Dataset. Nexus file containing the 23-taxa pruned tree with branch lengths.
(NEX)

File S4 Dataset. Excel file containing the raw mean values of the original 23 morphometric variables used in the diet-morphology correlation analyses. Diet data as used in the analyses are given in Table 1 of the main manuscript.
(XLS)

File S5 Dataset. Nexus file containing the topologies of the 166 taxon phylogeny from [16], the 350 and 635 taxon trees used in this paper and the binary matrices representing presence/absence of the Epibranchial lobe and mouthbrooding in each taxon for all trees.
(NEX)

File S6 Datafile. Raw genus-level morphometric data used in Principal Components Analyses to evaluate morphological differentiation at the genus level as illustrated in Figure 2 of the main paper.
(XLS)
