# Supplementary material for: Diet-Morphology Correlations in the Radiation of South American Geophagine Cichlids (Perciformes: Cichlidae: Cichlinae)
Source: PLoS One. 2012 Apr 2;7(4):e33997. doi: 10.1371/journal.pone.0033997 (PMC3317448; doi:10.1371/journal.pone.0033997)
Supplement: Table S1 — Species scores in a Principal Components Analysis and Procrustes Superimposition of 7 diet categories and 10 morphometric variables associated with feeding without phylogenetic correction. See text for explanation of the methods and Table 3 for rotation matrix and Procrustes rotation matrices for diet and morphology eigenvector values. (DOC) [file pone.0033997.s001.doc]

**S3**. Species scores in a Principal Components Analysis and Procrustes Superimposition of 7 diet categories and 10 morphometric variables associated with feeding without phylogenetic correction. See text for explanation of the methods and Table 3 for rotation matrix and Procrustes rotation matrices for diet and morphology eigenvector values.

|  | Morphology | | | | | | |  | Diet | | | | | | |
| --- | --- | --- | --- | --- | --- | --- | --- | --- | --- | --- | --- | --- | --- | --- | --- |
|  | PCA Scores | | |  | Procrustes PCA scores | | |  | PCA Scores | | |  | Procrustes rotated PCA scores | | |
|  | PC1 | PC2 | PC3 |  | PC1 | PC2 | PC3 |  | PC1 | PC2 | PC3 |  | PC1 | PC2 | PC3 |
| Eigenvalue | 4.117 | 2.005 | 1.158 |  |  |  |  |  | 2.184 | 1.572 | 1.449 |  |  |  |  |
| Cumulative percent variance explained | 41.2 | 61.2 | 72.8 |  |  |  |  |  | 31.2 | 53.7 | 74.4 |  |  |  |  |
|  |  |  |  |  |  |  |  |  |  |  |  |  |  |  |  |
| Species |  |  |  |  |  |  |  |  |  |  |  |  |  |  |  |
| *Apistogramma hoignei* | 0.290 | -1.043 | -0.822 |  | 0.036 | -0.128 | -0.101 |  | -0.056 | 0.963 | 0.488 |  | 0.086 | -0.079 | -0.064 |
| *Astronotus* sp. | 0.357 | -1.752 | -1.059 |  | 0.044 | -0.216 | -0.130 |  | 2.238 | 1.992 | 0.641 |  | 0.006 | -0.376 | -0.026 |
| *Biotodoma wavrini* | -0.202 | 1.247 | -0.242 |  | -0.025 | 0.154 | -0.030 |  | -0.857 | -0.807 | 0.216 |  | 0.044 | 0.134 | 0.041 |
| *Biotoecus dicentrarchus* | -0.762 | 0.281 | -2.894 |  | -0.094 | 0.035 | -0.356 |  | -0.609 | -1.058 | 0.317 |  | 0.033 | 0.126 | 0.084 |
| *Cichla orinocensis* | -0.530 | -0.427 | 0.125 |  | -0.065 | -0.053 | 0.015 |  | 0.989 | -0.183 | -2.213 |  | -0.293 | -0.018 | -0.060 |
| *Cichla temensis* | -1.075 | -0.320 | 0.849 |  | -0.132 | -0.039 | 0.105 |  | 0.606 | -0.503 | -2.514 |  | -0.313 | 0.050 | -0.069 |
| *Cichlasoma orinocense* | 0.751 | -1.456 | 0.540 |  | 0.092 | -0.179 | 0.067 |  | 0.944 | -0.111 | 1.051 |  | 0.053 | -0.111 | 0.124 |
| *Crenicichla geayi* | -1.493 | -0.057 | -0.560 |  | -0.184 | -0.007 | -0.069 |  | 1.917 | 0.415 | 1.510 |  | 0.063 | -0.255 | 0.155 |
| *Crenicichla* “O-lugubris” | -1.905 | -0.627 | -0.025 |  | -0.235 | -0.077 | -0.003 |  | 0.737 | -0.076 | -1.769 |  | -0.229 | -0.014 | -0.057 |
| *Crenicichla* “O-wallacii” | -1.840 | 0.323 | 1.379 |  | -0.227 | 0.040 | 0.170 |  | 0.729 | -0.298 | 0.633 |  | 0.015 | -0.065 | 0.105 |
| *Crenicichla sveni* | -1.284 | -0.169 | 0.128 |  | -0.158 | -0.021 | 0.016 |  | 0.880 | -0.370 | -0.975 |  | -0.164 | -0.029 | 0.025 |
| *Dicrossus filamentosus* | -0.710 | 0.597 | 0.692 |  | -0.087 | 0.074 | 0.085 |  | -0.774 | -1.278 | 0.126 |  | 0.015 | 0.164 | 0.084 |
| *Geophagus abalios* | 0.655 | 1.509 | -0.408 |  | 0.081 | 0.186 | -0.050 |  | -0.320 | 0.026 | 0.028 |  | 0.022 | 0.028 | -0.018 |
| *Geophagus dicrozoster* | 0.492 | 1.586 | -0.718 |  | 0.061 | 0.195 | -0.088 |  | -1.342 | 0.943 | -0.242 |  | 0.082 | 0.065 | -0.175 |
| *‘Geophagus’ steindachneri* | 1.302 | -0.156 | 0.729 |  | 0.160 | -0.019 | 0.090 |  | -0.106 | 0.272 | 0.580 |  | 0.075 | -0.026 | 0.003 |
| *Guianacara stergiosi* | 1.042 | -0.301 | -0.193 |  | 0.128 | -0.037 | -0.024 |  | -0.920 | 0.240 | 0.194 |  | 0.080 | 0.064 | -0.061 |
| *Gymnogeophagus australis* | 1.167 | 0.001 | -0.060 |  | 0.144 | 0.000 | -0.007 |  | -0.017 | -1.348 | 0.700 |  | 0.030 | 0.081 | 0.166 |
| *Hoplarchus psittacus* | 0.492 | -1.097 | -0.781 |  | 0.061 | -0.135 | -0.096 |  | -1.474 | 1.432 | -0.272 |  | 0.102 | 0.043 | -0.230 |
| *Mesonauta insignis* | 0.711 | -1.031 | 0.436 |  | 0.088 | -0.127 | 0.054 |  | -1.143 | 2.099 | -0.163 |  | 0.117 | -0.041 | -0.268 |
| *Mikrogeophagus ramirezi* | 0.040 | 0.588 | 1.312 |  | 0.005 | 0.072 | 0.161 |  | -0.107 | -1.211 | 0.643 |  | 0.033 | 0.081 | 0.145 |
| *Retroculus lapidifer* | 1.115 | -0.248 | 0.115 |  | 0.137 | -0.031 | 0.014 |  | -0.140 | -1.309 | 0.606 |  | 0.028 | 0.092 | 0.150 |
| *Satanoperca daemon* | 0.667 | 2.296 | -0.531 |  | 0.082 | 0.283 | -0.065 |  | -1.119 | 0.589 | -0.115 |  | 0.071 | 0.066 | -0.123 |
| *Satanoperca mapiritensis* | 0.721 | 0.255 | 1.990 |  | 0.089 | 0.031 | 0.245 |  | -0.056 | -0.421 | 0.531 |  | 0.045 | 0.021 | 0.068 |
