# Supplementary material for: Diet-Morphology Correlations in the Radiation of South American Geophagine Cichlids (Perciformes: Cichlidae: Cichlinae)
Source: PLoS One. 2012 Apr 2;7(4):e33997. doi: 10.1371/journal.pone.0033997 (PMC3317448; doi:10.1371/journal.pone.0033997)
Supplement: Table S2 — Species scores in a Principal Components Analysis of 7 diet categories and 10 morphometric variables associated with feeding with phylogenetic correction. Results are not given in the main text because correlations between diet and morphology disappear after phylogenetic correction. See text for details. (DOC) [file pone.0033997.s002.doc]

**S4**. Species scores in a Principal Components Analysis of 7 diet categories and 10 morphometric variables associated with feeding with phylogenetic correction. Results are not given in the main text because correlations between diet and morphology disappear after phylogenetic correction. See text for details.

|  | Morphology | | |  | Diet | | |
| --- | --- | --- | --- | --- | --- | --- | --- |
|  | PC1 | PC2 | PC3 |  | PC1 | PC2 | PC3 |
| Eigenvalue | 2.885 | 1.904 | 1.179 |  | 1.922 | 1.441 | 1.288 |
| Cumulative percent variance explained | 28.84 | 47.89 | 59.68 |  | 27.46 | 48.04 | 66.45 |
|  |  |  |  |  |  |  |  |
| Species |  |  |  |  |  |  |  |
| *Apistogramma hoignei* | -0.274 | -0.935 | -0.800 |  | -2.071 | -0.185 | 1.485 |
| *Astronotus* sp. | 2.006 | -0.128 | 1.660 |  | -0.655 | 0.185 | -1.467 |
| *Biotodoma wavrini* | -0.448 | 0.454 | 0.205 |  | -0.506 | 1.354 | -0.0552 |
| *Biotoecus dicentrarchus* | 0.485 | 1.862 | 0.868 |  | -0.260 | -0.344 | -1.150 |
| *Cichla orinocensis* | 0.248 | -1.377 | -0.785 |  | 0.307 | -0.760 | -0.589 |
| *Cichla temensis* | -0.150 | -1.251 | -0.641 |  | -0.361 | -0.880 | -0.081 |
| *Cichlasoma orinocense* | 0.636 | 1.126 | 0.256 |  | -0.069 | -0.619 | -1.368 |
| *Crenicichla geayi* | 0.203 | -1.122 | 0.058 |  | 0.565 | -0.516 | 0.227 |
| *Crenicichla* “O-lugubris” | -0.586 | 0.196 | -0.329 |  | -0.210 | -0.975 | 1.312 |
| *Crenicichla* “O-wallacii” | 0.005 | 2.230 | -1.45 |  | 0.921 | 0.548 | 1.081 |
| *Crenicichla sveni* | -1.039 | -0.901 | -0.203 |  | 0.479 | -0.239 | -0.389 |
| *Dicrossus filamentosus* | 2.550 | 0.047 | -1.055 |  | -1.061 | 0.283 | 0.669 |
| *Geophagus abalios* | 0.749 | -1.090 | -1.100 |  | -1.631 | 1.232 | 0.757 |
| *Geophagus dicrozoster* | -1.030 | 0.298 | 0.519 |  | -1.008 | -0.863 | 0.302 |
| *‘Geophagus’ steindachneri* | -0.803 | 0.352 | -1.267 |  | -0.491 | -0.135 | -0.724 |
| *Guianacara stergiosi* | -1.079 | 0.980 | -0.779 |  | -0.206 | -0.220 | -1.651 |
| *Gymnogeophagus australis* | 0.931 | -0.569 | 1.782 |  | 0.493 | 0.553 | -1.207 |
| *Hoplarchus psittacus* | 0.226 | 1.024 | 0.181 |  | 0.928 | -1.315 | -0.035 |
| *Mesonauta insignis* | -1.705 | -0.210 | 2.275 |  | 1.127 | 3.073 | 0.110 |
| *Mikrogeophagus ramirezi* | 0.216 | -0.042 | 0.483 |  | 1.770 | -1.133 | 1.800 |
| *Retroculus lapidifer* | -0.702 | -0.036 | 0.126 |  | 1.917 | 0.369 | 0.016 |
| *Satanoperca daemon* | -0.438 | -0.909 | -0.004 |  | -2.071 | -0.185 | 1.485 |
| *Satanoperca mapiritensis* | -0.274 | -0.935 | -0.800 |  | -0.655 | 0.185 | -1.467 |
