# Supplementary material for: Diet-Morphology Correlations in the Radiation of South American Geophagine Cichlids (Perciformes: Cichlidae: Cichlinae)
Source: PLoS One. 2012 Apr 2;7(4):e33997. doi: 10.1371/journal.pone.0033997 (PMC3317448; doi:10.1371/journal.pone.0033997)
Supplement: Table S3 — PCA Eigenvectors and individual scores for a dataset of 10 morphological variables for 207 individuals in 21 genera and 55 species of South American cichlids, including 15 geophagine genera. See text and Figure 2 for details. Institutional abbreviations are as follows: ANSP - Academy of Natural Sciences of Philadelphia, USA; FMNH: Field Museum of Natural History, USA; INPA: Fish collection of the Instituto Nacional de Pesquisas da Amazonia, Brazil; MCNG: Museo de Ciencias Naturales de Guanare, Venezuela; MCP: Museu de Zoologia do Universidad Pontificia do Rio Grande do Sul, Brazil; ROM: Royal Ontario Museum, Canada; UWI: Zoology collection of the University of the West Indies, Trinidad and Tobago. (DOC) [file pone.0033997.s003.doc]

**S5**. PCA Eigenvectors and individual scores for a dataset of 10 morphological variables for 207 individuals in 21 genera and 55 species of South American cichlids, including 15 geophagine genera. See text and Figure 2 for details. Institutional abbreviations are as follows: ANSP - Academy of Natural Sciences of Philadelphia, USA; FMNH: Field Museum of Natural History, USA; INPA: Fish collection of the Instituto Nacional de Pesquisas da Amazonia, Brazil; MCNG: Museo de Ciencias Naturales de Guanare, Venezuela; MCP: Museu de Zoologia do Universidad Pontificia do Rio Grande do Sul, Brazil; ROM: Royal Ontario Museum, Canada; UWI: Zoology collection of the University of the West Indies, Trinidad and Tobago.

|  | Morphology | | |
| --- | --- | --- | --- |
|  | PC1 | PC2 | PC3 |
| Eigenvalue | 5.938 | 1.665 | 1.000 |
| Cumulative percent variance explained | 59.38 | 76.03 | 86.03 |
|  |  |  |  |
| Species |  |  |  |
| LOG_SL | 0.000 | 0.000 | 1.000 |
| Head Length | 0.622 | -0.410 | -1.2E-09 |
| Heah Height | 0.973 | 0.174 | 1.06E-10 |
| Eye Position | 0.949 | 0.242 | -1E-10 |
| Eye Diameter | 0.873 | 0.143 | 2.78E-10 |
| Snout Length | 0.726 | 0.418 | -7.9E-10 |
| Body depth | 0.961 | -0.114 | 5.13E-10 |
| Peduncle depth | 0.776 | -0.563 | 3.01E-10 |
| Peduncle length, ventrally | -0.249 | 0.913 | -8.1E-10 |
| Mouth position | 0.909 | 0.222 | 1.67E-10 |

|  | Species | Catalogue number | Locality | SL | PC1 | PC2 | PC3 |
| --- | --- | --- | --- | --- | --- | --- | --- |
| 1 | *Acarichthys heckelii* | ROM 85767 | Pirara river, Guyana | 77.2 | 0.741 | 0.652 | -0.209 |
| 2 | *Acarichthys heckelii* | ROM 85767 | Pirara river, Guyana | 65.9 | 0.715 | 0.873 | -0.452 |
| 3 | *Acarichthys heckelii* | ROM 85767 | Pirara river, Guyana | 75.3 | 0.554 | 1.028 | -0.246 |
| 4 | *Acarichthys heckelii* | ROM 85767 | Pirara river, Guyana | 74.8 | 0.740 | 0.783 | -0.256 |
| 5 | *Acarichthys heckelii* | ROM 85767 | Pirara river, Guyana | 71.4 | 0.656 | 0.903 | -0.328 |
| 6 | *Apistogramma cacatuoides* | ROM 56411 | Amazonas river, Colombia | 40.0 | 0.251 | -0.253 | -1.218 |
| 7 | *Apistogramma cacatuoides* | ROM 56411 | Amazonas river, Colombia | 38.3 | 0.403 | -0.252 | -1.287 |
| 8 | *Apistogramma cf steindachneri* | ROM 85746 | Rupununi savannas, Guyana | 31.4 | 0.360 | -0.395 | -1.590 |
| 9 | *Apistogramma eunotus* | ROM 56457 | Amazonas river, Colombia | 32.1 | 0.074 | 0.172 | -1.558 |
| 10 | *Apistogramma eunotus* | ROM 56457 | Amazonas river, Colombia | 27.8 | 0.009 | -0.217 | -1.778 |
| 11 | *Apistogramma eunotus* | ROM 56457 | Amazonas river, Colombia | 41.9 | 0.103 | -0.523 | -1.146 |
| 12 | *Apistogramma hoignei* | MCNG13636 | Caño El Pozuelo, Venezuela | 29.3 | -0.050 | -0.651 | -1.694 |
| 13 | *Apistogramma hoignei* | MCNG28647 | Caño Maporal, Venezuela | 29.4 | -0.021 | -0.540 | -1.690 |
| 14 | *Apistogramma hoignei* | MCNG28566 | Caño Caicara, Venezuela | 34.0 | -0.148 | -0.084 | -1.470 |
| 15 | *Apistogramma hoignei* | MCNG37302 | Caura river, Venezuela | 28.1 | 0.024 | 0.010 | -1.759 |
| 16 | *Apistogramma sp "spot up"* | ROM 86002 | Rupununi river, Guyana | 31.6 | 0.486 | -0.607 | -1.583 |
| 17 | *Apistogramma sp "spot up"* | ROM 86002 | Rupununi river, Guyana | 33.6 | 0.311 | -0.334 | -1.484 |
| 18 | *Apistogramma sp "spot up"* | ROM 86002 | Rupununi river, Guyana | 28.4 | 0.501 | -0.792 | -1.744 |
| 19 | *Apistogramma urteagai* | ROM 76893 | Madre de Dios, Peru | 38.3 | 0.249 | -0.442 | -1.286 |
| 20 | *Apistogramma urteagai* | ROM 76893 | Madre de Dios, Peru | 35.6 | 0.203 | -0.514 | -1.396 |
| 21 | *Apistogramma urteagai* | ROM 82787 | Madre de Dios, Peru | 29.3 | 0.189 | -0.435 | -1.697 |
| 22 | *Apistogramma urteagai* | ROM 82787 | Madre de Dios, Peru | 26.3 | 0.199 | -0.276 | -1.860 |
| 23 | *Astronotus ocellatus* | ROM 71987 | Ontario introduced | 149.1 | 0.887 | -1.364 | 0.803 |
| 24 | *Astronotus ocellatus* | ROM 55447 | Yarina Cocha, Peru | 99.7 | 0.526 | -1.679 | 0.184 |
| 25 | *Astronotus sp* | MCNG4994 | Fernando Corrales module, Venezuela | 192.2 | 0.545 | -1.362 | 1.193 |
| 26 | *Astronotus sp* | MCNG1675 | Arismendi, Barinas, Venezuela | 203.4 | 0.565 | -2.156 | 1.280 |
| 27 | *Astronotus sp* | MCNG43792 | Caño Caicara, Venezuela | 178.1 | 0.011 | -1.503 | 1.076 |
| 28 | *Astronotus sp* | MCNG5921 | Caño Uvericas, Venezuela | 199.4 | 0.335 | -1.585 | 1.250 |
| 29 | *Astronotus sp* | MCNG10112 | Caño Maporal, Venezuela | 222.5 | 0.519 | -1.607 | 1.418 |
| 30 | *Biotodoma cupido* | ROM 85769 | Rupununi river, Guyana | 97.2 | 0.683 | 0.976 | 0.146 |
| 31 | *Biotodoma cupido* | ROM 85769 | Rupununi river, Guyana | 93.2 | 0.712 | 0.777 | 0.081 |
| 32 | *Biotodoma wavrini* | MCNG38330 | Manipitare river, Venezuela | 98.8 | 0.713 | 0.928 | 0.171 |
| 33 | *Biotodoma wavrini* | MCNG22905 | Ventuari river, Venezuela | 99.5 | 0.001 | 0.905 | 0.181 |
| 34 | *Biotodoma wavrini* | MCNG20037 | Caño La Pica, Venezuela | 84.9 | 0.120 | 0.962 | -0.062 |
| 35 | *Biotodoma wavrini* | MCNG38645 | Emoni river, Venezuela | 97.8 | 0.461 | 0.888 | 0.154 |
| 36 | *Biotodoma wavrini* | MCNG37230 | Caño Duapo, Venezuela | 107.2 | 0.173 | 0.946 | 0.295 |
| 37 | *Biotoecus dicentrarchus* | MCNG21792 | Cinaruco river, Venezuela | 33.5 | -0.659 | 1.517 | -1.493 |
| 38 | *Biotoecus dicentrarchus* | MCNG33212 | Paso Acosta lagoon, Venezuela | 35.0 | -1.202 | 1.876 | -1.425 |
| 39 | *Biotoecus dicentrarchus* | MCNG33212 | Paso Acosta lagoon, Venezuela | 34.9 | -1.153 | 1.932 | -1.426 |
| 40 | *Biotoecus dicentrarchus* | MCNG39950 | Cinaruco river, Venezuela | 30.6 | -1.007 | 1.376 | -1.628 |
| 41 | *Biotoecus dicentrarchus* | MCNG39950 | Cinaruco river, Venezuela | 29.2 | -1.396 | 1.387 | -1.704 |
| 42 | *Cichla melaniae* | ROM 84283 | Iriri river, Brazil | 195.7 | -0.579 | 0.470 | 1.221 |
| 43 | *Cichla ocellaris* | ROM 86140 | Pirara river, Guyana | 223.1 | -0.766 | 0.313 | 1.422 |
| 44 | *Cichla ocellaris* | ROM 86140 | Pirara river, Guyana | 215.1 | -0.993 | 0.374 | 1.366 |
| 45 | *Cichla ocellaris* | ROM 85738 | Rupununi river, Guyana | 199.1 | -0.992 | 0.518 | 1.247 |
| 46 | *Cichla ocellaris* | ROM 86212 | Rupununi river, Guyana | 222.7 | -0.689 | 0.313 | 1.419 |
| 47 | *Cichla ocellaris* | ROM 86212 | Rupununi river, Guyana | 197.1 | -0.789 | 0.272 | 1.232 |
| 48 | *Cichla orinocensis* | MCNG43956 | Caño Caicara, Venezuela | 209.4 | -0.604 | 0.362 | 1.324 |
| 49 | *Cichla orinocensis* | MCNG1769 | Caño Macaurel, Venezuela | 188.6 | -0.825 | -0.199 | 1.163 |
| 50 | *Cichla temensis* | ROM 86127 | Pirara river, Guyana | 226.3 | -1.214 | 0.486 | 1.444 |
| 51 | *Cichla temensis* | MCNG12265 | Siapa river, Venezuela | 229.5 | -1.180 | 0.538 | 1.465 |
| 52 | *Cichla temensis* | MCNG1857 | Hato santiago, Venezuela | 225.0 | -0.883 | 0.122 | 1.435 |
| 53 | *Cichlasoma amazonarum* | ROM 56322 | Amazonas river, Colombia | 66.6 | 1.022 | -1.616 | -0.436 |
| 54 | *Cichlasoma amazonarum* | ROM 55455 | Yarina Cocha, Peru | 51.1 | 1.120 | -1.480 | -0.843 |
| 55 | *Cichlasoma amazonarum* | ROM 83951 | Cocha Cashu, Manu, Peru | 86.7 | 1.097 | -1.318 | -0.029 |
| 56 | *Cichlasoma amazonarum* | ROM 82893 | Rio Tambopata, Peru | 63.6 | 1.242 | -1.323 | -0.505 |
| 57 | *Cichlasoma amazonarum* | ROM 56322 | Amazonas river, Colombia | 79.8 | 0.878 | -1.362 | -0.157 |
| 58 | *Cichlasoma bimaculatum* | ROM 66564 | Moruka-Pomeroon rivers, Guyana | 78.0 | 1.015 | -1.396 | -0.192 |
| 59 | *Cichlasoma bimaculatum* | ROM 66564 | Moruka-Pomeroon rivers, Guyana | 86.6 | 0.888 | -1.171 | -0.031 |
| 60 | *Cichlasoma bimaculatum* | ROM 66564 | Moruka-Pomeroon rivers, Guyana | 89.7 | 0.710 | -1.283 | 0.022 |
| 61 | *Cichlasoma bimaculatum* | ROM 36564 | Trenches around Georgetown, Guyana | 116.2 | 1.076 | -1.465 | 0.420 |
| 62 | *Cichlasoma bimaculatum* | ROM 36564 | Trenches around Georgetown, Guyana | 88.8 | 0.844 | -1.073 | 0.006 |
| 63 | *Cichlasoma boliviense* | ROM 76285 | Rio Madre de Dios, Peru | 78.8 | 1.038 | -1.138 | -0.177 |
| 64 | *Cichlasoma boliviense* | ROM 76289 | Rio Beni, Peru | 90.2 | 0.846 | -1.499 | 0.030 |
| 65 | *Cichlasoma boliviense* | ROM 76289 | Rio Beni, Peru | 81.6 | 1.227 | -1.612 | -0.123 |
| 66 | *Cichlasoma orinocense* | MCNG37617 | Caño Caicara, Venezuela | 77.8 | 0.768 | -2.086 | -0.196 |
| 67 | *Cichlasoma orinocense* | MCNG7132 | Socopo Viejo river, Venezuela | 100.4 | 1.063 | -1.522 | 0.196 |
| 68 | *Cichlasoma orinocense* | MCNG26950 | Caño El Rosario, Venezuela | 76.3 | 0.755 | -2.075 | -0.226 |
| 69 | *Cichlasoma orinocense* | MCNG16799 | Caño S of El Pilar, Venezuela | 62.8 | 1.016 | -1.297 | -0.524 |
| 70 | *Cichlasoma orinocense* | MCNG13944 | Prestamo near Mantecal, Venezuela | 88.5 | 1.002 | -2.115 | 0.002 |
| 71 | *Crenicichla geayi* | MCNG13787 | Chorreron river, Venezuela | 106.1 | -1.947 | -0.716 | 0.281 |
| 72 | *Crenicichla geayi* | MCNG13801 | Caño near Apartaderos, Venezuela | 93.9 | -1.752 | -0.742 | 0.093 |
| 73 | *Crenicichla geayi* | MCNG14447 | Caño Jobal, Venezuela | 112.4 | -1.534 | -1.005 | 0.369 |
| 74 | *Crenicichla geayi* | MCNG27298 | Caño Maraca, Venezuela | 133.9 | -1.067 | -1.426 | 0.638 |
| 75 | *Crenicichla geayi* | MCNG6482 | Quinimari river, Venezuela | 129.2 | -1.890 | -0.864 | 0.582 |
| 76 | *Crenicichla sveni* | MCNG4517 | Caño Caripito, Venezuela | 107.1 | -1.495 | -0.829 | 0.295 |
| 77 | *Crenicichla sveni* | MCNG1768 | Caño Macaurel, Venezuela | 122.1 | -1.645 | -0.916 | 0.496 |
| 78 | *Crenicichla sveni* | MCNG13550 | Caño Caicara, Venezuela | 138.9 | -1.065 | -0.990 | 0.694 |
| 79 | *Crenicichla sveni* | MCNG7990 | Caño Bucarito, Venezuela | 122.5 | -1.469 | -1.008 | 0.501 |
| 80 | *Crenicichla sveni* | MCNG18623 | Socopo Viejo river, Venezuela | 206.1 | -1.366 | -0.907 | 1.300 |
| 81 | *Crenicichla cf alta* | ROM 86057 | Rupununi savannas, Guyana | 136.6 | -1.136 | -1.044 | 0.669 |
| 82 | *Crenicichla cf alta* | ROM 86057 | Rupununi savannas, Guyana | 116.4 | -1.423 | -0.948 | 0.423 |
| 83 | *Crenicichla frenata* | UWI 96.08.2 | Cunupia, Trinidad and Tobago | 205.0 | -1.479 | -1.201 | 1.292 |
| 84 | *Crenicichla frenata* | UWI 96.01.30 | Matura river, Trinidad and Tobago | 150.0 | -1.653 | -1.126 | 0.812 |
| 85 | *Crenicichla frenata* | UWI 96.8.15 | Tompure river, Trinidad and Tobago | 150.4 | -1.615 | -0.800 | 0.816 |
| 86 | *Crenicichla frenata* | UWI 73.4.2 | Curamata river, Trinidad and Tobago | 164.3 | -1.695 | -1.441 | 0.952 |
| 87 | *Crenicichla frenata* | UWI 96.06.27 | Santa Cruz river, Trinidad and Tobago | 150.5 | -1.665 | -1.070 | 0.817 |
| 88 | *Crenicichla lepidota* | ROM 67355 | Tagatija-Mi, Paraguay | 92.1 | -1.020 | -0.776 | 0.063 |
| 89 | *Crenicichla Orinoco-lugubris* | MCNG22877 | Caño Tabaro, Venezuela | 217.4 | -1.649 | -0.824 | 1.382 |
| 90 | *Crenicichla Orinoco-lugubris* | MCNG40290 | Cinaruco river, Venezuela | 184.7 | -2.052 | -0.587 | 1.132 |
| 91 | *Crenicichla Orinoco-lugubris* | MCNG10998 | Caño Agua Blanca, Venezuela | 231.0 | -1.510 | -0.913 | 1.475 |
| 92 | *Crenicichla Orinoco-lugubris* | MCNG40251 | Cinaruco river, Venezuela | 233.2 | -1.769 | -0.329 | 1.490 |
| 93 | *Crenicichla Orinoco-lugubris* | MCNG26453 | Cinaruco river, Venezuela | 183.1 | -1.970 | -0.404 | 1.118 |
| 94 | *Crenicichla Orinoco-wallacii* | MCNG30065 | Guri camp, Venezuela | 56.9 | -2.172 | 0.468 | -0.677 |
| 95 | *Crenicichla Orinoco-wallacii* | MCNG38137 | Caño Buridajow, Venezuela | 44.3 | -2.033 | 0.397 | -1.062 |
| 96 | *Crenicichla Orinoco-wallacii* | MCNG40952 | Cinaruco river, Venezuela | 51.5 | -2.263 | 0.510 | -0.831 |
| 97 | *Crenicichla Orinoco-wallacii* | MCNG4513 | Caño W of Buena Vista, Venezuela | 49.0 | -1.943 | 0.362 | -0.907 |
| 98 | *Crenicichla Orinoco-wallacii* | MCNG4509 | Riecito river, Venezuela | 46.6 | -2.397 | 0.201 | -0.985 |
| 99 | *Crenicichla reticulata* | ANSP179089 | Rupununi river, Guyana | 85.8 | -1.337 | -0.599 | -0.045 |
| 100 | *Crenicichla reticulata* | ANSP 179090 | Rupununi river, Guyana | 78.1 | -1.426 | -0.622 | -0.190 |
| 101 | *Crenicichla saxatilis* | ROM66575 | Waini river, Guyana | 123.1 | -1.551 | -0.739 | 0.508 |
| 102 | *Crenicichla saxatilis* | ROM 66544 | Waini river, Guyana | 136.9 | -1.367 | -1.130 | 0.671 |
| 103 | *Crenicichla saxatilis* | ROM 22105 | Onverwagt, Guyana | 125.3 | -1.365 | -1.045 | 0.536 |
| 104 | *Crenicichla saxatilis* | ROM 22105 | Onverwagt, Guyana | 122.3 | -1.667 | -0.956 | 0.499 |
| 105 | *Crenicichla sedentaria* | ROM 55468 | Llullapichis drainage, Peru | 78.3 | -1.550 | -0.335 | -0.187 |
| 106 | *Crenicichla sedentaria* | ROM 55468 | Llullapichis drainage, Peru | 63.5 | -1.572 | -0.235 | -0.507 |
| 107 | *Crenicichla sedentaria* | ROM 55463 | Llullapichis drainage, Peru | 72.7 | -2.029 | -0.672 | -0.301 |
| 108 | *Crenicichla sedentaria* | ROM 55466 | Huambo river, Peru | 60.9 | -2.071 | -0.188 | -0.572 |
| 109 | *Crenicichla sedentaria* | ROM 55466 | Huambo river, Peru | 60.3 | -1.910 | -0.169 | -0.587 |
| 110 | *Crenicichla semicincta* | ROM 84055 | Rio Manu, Peru | 74.3 | -1.017 | -0.787 | -0.267 |
| 111 | *Dicrossus filamentosus* | MCNG42033 | Caño Buridajow, Venezuela | 32.4 | -1.046 | 0.783 | -1.541 |
| 112 | *Dicrossus filamentosus* | MCNG35175 | Pasiba river, Venezuela | 28.9 | -0.580 | 0.940 | -1.717 |
| 113 | *Dicrossus filamentosus* | MCNG35175 | Pasiba river, Venezuela | 27.8 | -0.659 | 1.116 | -1.780 |
| 114 | *Dicrossus filamentosus* | MCNG42298 | Pasimoni river, Venezuela | 34.7 | -0.875 | 1.232 | -1.438 |
| 115 | *Dicrossus filamentosus* | MCNG42298 | Pasimoni river, Venezuela | 29.9 | -0.760 | 0.952 | -1.667 |
| 116 | *Dicrossus maculatus* | ROM Uncatalogued | Aquarium | 30.0 | -0.488 | 0.793 | -1.661 |
| 117 | *Dicrossus maculatus* | ROM Uncatalogued | Aquarium | 28.8 | -0.136 | 0.959 | -1.725 |
| 118 | *Dicrossus maculatus* | ROM Uncatalogued | Aquarium | 31.8 | -0.249 | 0.573 | -1.573 |
| 119 | *Dicrossus maculatus* | ROM Uncatalogued | Aquarium | 28.4 | -0.568 | 0.669 | -1.742 |
| 120 | *Dicrossus maculatus* | ROM Uncatalogued | Aquarium | 26.2 | -0.150 | 1.317 | -1.867 |
| 121 | *Geophagus abalios* | MCNG33851 | Aricagua lagoon, Venezuela | 161.4 | 0.621 | 1.303 | 0.924 |
| 122 | *Geophagus abalios* | MCNG31133 | Orisnera lagoon, Venezuela | 137.7 | 0.464 | 1.507 | 0.680 |
| 123 | *Geophagus abalios* | MCNG37650 | Cinaruco river, Venezuela | 181.4 | 0.286 | 1.471 | 1.104 |
| 124 | *Geophagus abalios* | MCNG30680 | Aguaro river, Venezuela | 201.1 | 0.503 | 1.492 | 1.263 |
| 125 | *Geophagus abalios* | MCNG33211 | Paso Acosta lagoon, Venezuela | 150.1 | 0.642 | 1.255 | 0.814 |
| 126 | *Geophagus altifrons* | INPA 14698 | Uatumã river, Brazil | 189.8 | 0.688 | 1.461 | 1.173 |
| 127 | *Geophagus altifrons* | INPA 14698 | Uatumã river, Brazil | 187.5 | 0.789 | 1.261 | 1.155 |
| 128 | *Geophagus altifrons* | INPA 14698 | Uatumã river, Brazil | 192.6 | 0.708 | 1.314 | 1.196 |
| 129 | *Geophagus altifrons* | INPA 14698 | Uatumã river, Brazil | 185.5 | 0.545 | 1.500 | 1.138 |
| 130 | *Geophagus altifrons* | INPA 14698 | Uatumã river, Brazil | 168.4 | 0.676 | 1.393 | 0.989 |
| 131 | *Geophagus dicrozoster* | MCNG21599 | Nichare river, Venezuela | 184.7 | 0.668 | 1.318 | 1.132 |
| 132 | *Geophagus dicrozoster* | MCNG36629 | Caura river, Venezuela | 163.4 | 0.440 | 1.433 | 0.944 |
| 133 | *Geophagus dicrozoster* | MCNG41487 | Cinaruco river, Venezuela | 160.1 | 0.249 | 1.562 | 0.912 |
| 134 | *Geophagus dicrozoster* | MCNG36808 | Caroni river, Venezuela | 158.3 | 0.072 | 1.637 | 0.895 |
| 135 | *Geophagus dicrozoster* | MCNG20143 | Cinaruco river, Venezuela | 198.2 | 0.227 | 1.494 | 1.240 |
| 136 | *Geophagus harreri* | ANSP 187130 | Lawa river, Suriname | 124.9 | 0.799 | 1.037 | 0.531 |
| 137 | *Geophagus harreri* | ANSP 187136 | Litanie river, Suriname | 166.5 | 0.839 | 0.941 | 0.973 |
| 138 | *Geophagus winemilleri* | INPA 12062 | Jaú river, Brazil | 215.2 | 0.320 | 1.569 | 1.367 |
| 139 | *Geophagus winemilleri* | INPA 3168 | Vaupés river, Brazil | 205.1 | 0.557 | 1.112 | 1.292 |
| 140 | *Geophagus winemilleri* | INPA 3176 | Rio Negro, Brazil | 181.8 | 0.417 | 1.613 | 1.107 |
| 141 | *Geophagus winemilleri* | INPA 4936 | Rio Negro, Brazil | 193.2 | 0.494 | 1.324 | 1.201 |
| 142 | *Geophagus winemilleri* | INPA 4936 | Rio Negro, Brazil | 186.9 | 0.521 | 1.499 | 1.150 |
| 143 | *Geophagus brasiliensis* | MCP 18976 | Uruguai river, Brazil | 136.9 | 0.482 | 0.247 | 0.672 |
| 144 | *Geophagus brasiliensis* | MCP 18976 | Uruguai river, Brazil | 128.8 | 0.606 | 0.523 | 0.578 |
| 145 | *Geophagus brasiliensis* | MCP 18976 | Uruguai river, Brazil | 130.9 | 0.552 | 0.426 | 0.603 |
| 146 | *Geophagus crassilabris* | FMNH 29188 | Upper Rio Chagres, Panama | 154.7 | 0.611 | 0.371 | 0.860 |
| 147 | *Geophagus crassilabris* | FMNH 29188 | Upper Rio Chagres, Panama | 112.3 | 0.411 | 0.680 | 0.367 |
| 148 | *Geophagus crassilabris* | FMNH 8142 | Canal Zone reservoir creek, Panama | 104.1 | 0.687 | 0.685 | 0.251 |
| 149 | *Geophagus crassilabris* | FMNH 8139 | Rio Frijoles, Panama | 98.1 | 0.537 | 0.679 | 0.160 |
| 150 | *Geophagus crassilabris* | FMNH 8140 | Rio Mandingo, Panama | 91.5 | 0.542 | 0.356 | 0.053 |
| 151 | *Geophagus pellegrini* | FMNH 58594 | Istmina, Colombia | 103.0 | 0.682 | 0.689 | 0.235 |
| 152 | *Geophagus pellegrini* | FMNH 58594 | Istmina, Colombia | 82.2 | 0.412 | 0.618 | -0.113 |
| 153 | *Geophagus pellegrini* | FMNH 58587 | Boca de Certegai, Colombia | 109.7 | 0.790 | 0.699 | 0.332 |
| 154 | *Geophagus pellegrini* | FMNH 58587 | Boca de Certegai, Colombia | 108.6 | 0.642 | 0.613 | 0.316 |
| 155 | *‘Geophagus’ steindachneri* | FMNH 58582 | Soplaviento, Colombia | 96.0 | 0.600 | 0.169 | 0.126 |
| 156 | *Geophagus’ steindachneri* | MCNG33384 | Chachiri river, Venezuela | 80.4 | 0.803 | 0.015 | -0.147 |
| 157 | *Geophagus’ steindachneri* | MCNG33384 | Chachiri river, Venezuela | 83.9 | 1.111 | -0.204 | -0.081 |
| 158 | *Guianacara stergiosi* | MCNG34395 | Caroni river, Venezuela | 70.2 | 1.277 | 0.661 | -0.354 |
| 159 | *Guianacara stergiosi* | MCNG34152 | Guri dam, Venezuela | 67.7 | 0.991 | 0.351 | -0.411 |
| 160 | *Guianacara stergiosi* | MCNG36989 | El Espiritu river, Venezuela | 69.0 | 0.903 | 0.740 | -0.381 |
| 161 | *Guianacara stergiosi* | MCNG34152 | Guri dam, Venezuela | 65.3 | 0.942 | 0.602 | -0.465 |
| 162 | *Guianacara stergiosi* | MCNG30017 | Guri camp, Venezuela | 60.2 | 0.915 | 0.717 | -0.591 |
| 163 | *Guianacara takutu* | ROM 85721 | Pirara river, Guyana | 57.0 | 0.874 | 0.543 | -0.674 |
| 164 | *Guianacara takutu* | ROM 85721 | Pirara river, Guyana | 55.0 | 0.933 | 0.153 | -0.730 |
| 165 | *Guianacara takutu* | ROM 85721 | Pirara river, Guyana | 57.8 | 0.810 | 0.519 | -0.653 |
| 166 | *Gymnogeophagus rhabdotus* | MCP 18662 | Rio Jacui, Brazil | 70.5 | 0.854 | 0.122 | -0.347 |
| 167 | *Gymnogeophagus rhabdotus* | MCP 18662 | Rio Jacui, Brazil | 80.2 | 0.985 | 0.022 | -0.150 |
| 168 | *Gymnogeophagus rhabdotus* | MCP 18662 | Rio Jacui, Brazil | 76.8 | 1.100 | -0.052 | -0.217 |
| 169 | *Gymnogeophagus rhabdotus* | MCP 9016 | Rio Jacui, Brazil | 73.9 | 0.827 | -0.028 | -0.276 |
| 170 | *Gymnogeophagus rhabdotus* | MCP 9016 | Rio Jacui, Brazil | 85.7 | 0.910 | -0.013 | -0.047 |
| 171 | *Hoplarchus psittacus* | MCNG21786 | Cinaruco river, Venezuela | 187.0 | 0.893 | 0.264 | 1.151 |
| 172 | *Hoplarchus psittacus* | MCNG40997 | Cinaruco river, Venezuela | 217.7 | 0.858 | 0.005 | 1.384 |
| 173 | *Hoplarchus psittacus* | MCNG1886 | Aguaro river, Venezuela | 194.7 | 0.942 | -0.368 | 1.213 |
| 174 | *Hoplarchus psittacus* | MCNG26455 | Cinaruco river, Venezuela | 145.1 | 1.091 | 0.013 | 0.761 |
| 175 | *Hoplarchus psittacus* | MCNG18770 | Las Majaguas dam, Venezuela | 169.9 | 0.874 | -0.366 | 1.003 |
| 176 | *Mazarunia mazaruni* | ROM 83869 | Mazaruni river, Guyana | 47.6 | 0.120 | 0.060 | -0.951 |
| 177 | *Mazarunia mazaruni* | ROM 83869 | Mazaruni river, Guyana | 47.4 | 0.084 | 0.234 | -0.956 |
| 178 | *Mazarunia mazaruni* | ROM 83869 | Mazaruni river, Guyana | 39.8 | 0.175 | 0.280 | -1.227 |
| 179 | *Mesonauta egregius* | MCNG4459 | Fernando Corrales module, Venezuela | 63.1 | 1.446 | -2.208 | -0.518 |
| 180 | *Mesonauta egregius* | MCNG7142 | Socopo Viejo river, Venezuela | 66.3 | 1.291 | -2.175 | -0.441 |
| 181 | *Mesonauta egregius* | MCNG30443 | Caroni river, Venezuela | 66.3 | 1.198 | -1.676 | -0.441 |
| 182 | *Mesonauta egregius* | MCNG31725 | Aguas Muertas river, Venezuela | 65.2 | 1.431 | -2.128 | -0.469 |
| 183 | *Mesonauta insignis* | ROM 84976 | Rio Cinaruco, Venezuela | 79.0 | 0.971 | -1.517 | -0.174 |
| 184 | *Mesonauta insignis* | ROM 84976 | Rio Cinaruco, Venezuela | 61.0 | 1.337 | -1.785 | -0.571 |
| 185 | *Mikrogeophagus ramirezi* | MCNG28191 | Caño Maporal, Venezuela | 34.0 | 0.260 | 0.162 | -1.467 |
| 186 | *Mikrogeophagus ramirezi* | MCNG42966 | Near Mantecal, Venezuela | 30.5 | 0.501 | 0.040 | -1.637 |
| 187 | *Mikrogeophagus ramirezi* | MCNG32837 | Aguaro river, Venezuela | 32.6 | 0.283 | 0.474 | -1.532 |
| 188 | *Mikrogeophagus ramirezi* | MCNG14350 | Caño near Calabozo, Venezuela | 29.8 | 0.160 | 0.394 | -1.671 |
| 189 | *Mikrogeophagus ramirezi* | MCNG37193 | Agua Linda lagoon, Venezuela | 32.6 | -0.047 | 0.460 | -1.532 |
| 190 | *Retroculus lapidifer* | ROM 84142 | Urauaçu, Brazil | 95.8 | 0.348 | 0.621 | 0.123 |
| 191 | *Retroculus lapidifer* | ROM 84142 | Urauaçu, Brazil | 101.3 | -0.028 | 0.721 | 0.209 |
| s | *Retroculus lapidifer* | ROM 84145 | Rio das Mortes, Brazil | 145.0 | 0.271 | 0.886 | 0.759 |
| 193 | *Satanoperca daemon* | MCNG37138 | Tauca river, Venezuela | 157.6 | 0.522 | 0.476 | 0.888 |
| 194 | *Satanoperca daemon* | MCNG20142 | Cinaruco river, Venezuela | 190.2 | 0.400 | 0.562 | 1.177 |
| 195 | *Satanoperca daemon* | MCNG28407 | Rio Negro, Venezuela | 143.0 | 0.332 | 0.809 | 0.739 |
| 196 | *Satanoperca daemon* | MCNG3301 | Manapiare river, Venezuela | 174.8 | 0.563 | 0.293 | 1.047 |
| 197 | *Satanoperca daemon* | MCNG31916 | Aguaro river, Venezuela | 122.9 | 0.494 | 0.661 | 0.506 |
| 198 | *Satanoperca jurupari* | INPA 14686 | Uatumã river, Brazil | 207.6 | 0.530 | 0.826 | 1.311 |
| 199 | *Satanoperca jurupari* | INPA 14686 | Uatumã river, Brazil | 159.2 | 0.564 | 0.529 | 0.904 |
| 200 | *Satanoperca mapiritensis* | MCNG33270 | El Potrero lagoon, Venezuela | 137.9 | 0.517 | 0.557 | 0.682 |
| 201 | *Satanoperca mapiritensis* | MCNG10119 | Caño Maporal, Venezuela | 164.3 | 0.749 | 0.563 | 0.952 |
| 202 | *Satanoperca mapiritensis* | MCNG11123 | Caño Garrapata, Venezuela | 138.1 | 0.594 | 0.440 | 0.685 |
| 203 | *Satanoperca mapiritensis* | MCNG7135 | Socopo Viejo river, Venezuela | 140.6 | 0.642 | 0.784 | 0.713 |
| 204 | *Satanoperca mapiritensis* | MCNG31382 | Paramuto lagoon, Venezuela | 154.6 | 0.575 | 0.721 | 0.858 |
| 205 | *Taeniacara candidi* | ROM Uncatalogued | Aquarium | 29.8 | -1.127 | 0.562 | -1.668 |
| 206 | *Taeniacara candidi* | ROM Uncatalogued | Aquarium | 30.1 | -0.900 | 0.607 | -1.654 |
| 207 | *Taeniacara candidi* | ROM Uncatalogued | Aquarium | 31.1 | -1.005 | 0.405 | -1.605 |
