# Supplementary material for: Diet-Morphology Correlations in the Radiation of South American Geophagine Cichlids (Perciformes: Cichlidae: Cichlinae)
Source: PLoS One. 2012 Apr 2;7(4):e33997. doi: 10.1371/journal.pone.0033997 (PMC3317448; doi:10.1371/journal.pone.0033997)
Supplement: File S1 — Description of morphometric measurements taken in this study. The fist list includes the intial 23 variables measured in the comparative study of morphology and diet correlations. The second list shows the measures used in the comparison of intrageneric versus intergeneric morphometric variation illustrated in Fig. 2. (DOC) [file pone.0033997.s004.doc]

S1 – Description of morphometric measurements taken in this study. The fist list includes the intial 23 variables measured in the comparative study of morphology and diet correlations. The second list shows the measures used in the comparison of intrageneric versus intergeneric morphometric variation illustrated in Fig. 2

1 - Standard Length (SL): Distance between the tip of the upper lip with mouth completely closed to the midpoint of the caudal peduncle where the caudal fin rays insert into the hypural plates.

2 - Head length: Measured from the tip of the upper lip with the mouth completely closed to the caudal edge of the operculum.

3 - Head height: The vertical distance through the center of the eye, between the dorsal and ventral edges of the head.

4 - Gape width: the horizontal internal distance between the tips of the premaxilla with the mouth fully open and protruded.

5 - Interorbital distance: the distance between the dorsal bony edge of the orbit on either side of the head.

6 - Eye position: the vertical distance between the center of the eye and the ventral edge of the head.

7 - Eye diameter: the longest horizontal distance between the anterior and posterior edges of the eye.

8 - Snout length, mouth closed: the distance from the center of the eye to the center the upper lip (i.e. the symphysis of the premaxilla).

9 - Snout length, mouth protruded: same as above, but with the mouth fully protruded.

10 – Mouth protrusion: 9 minus 8

11 - Mouth position: Estimated by drawing a reference horizontal line that passes from the corner of the mouth to the middle of the insertion of caudal rays in the caudal peduncle. With the fish fixed in this position, a point is found equivalent to the anterior-most point of the orbit, and another corresponding to the tip of the upper lip. A line was traced between the two points and the angle between the two measured.

12 - Ceratobranchial length: taken on dissected first ceratobranchial. The straight distance between the joint of the basibranchial with the ceratobranchial and the joint between the ceratobranchial and the epibranchial.

13 - Ceratobranchial gill-raker number: number of rakers between the anterior tip of the ceratobranchial and the corner of the epibranchial, including the one in the corner (but not the one(s) on the epibranchial.

14 - Ceratobranchial inter gill-raker space: the average distance between ceratobranchial gill rakers from five measurements.

15 - Ceratobranchial gill-raker length: the average distance between the base of five gill rakers and their tip.

16 - Epibranchial lobe length: in geophagines with epibranchial lobe, the longest distance between the base of the lobe and its tip, excluding gill rakers.

17 - Epibranchial lobe depth: the longest distance between the dorsal and ventral edges of the lobe, excluding gill rakers.

18 - Epibranchial gill raker number: number of rakers in the epibranchial.

19 - Epibranchial inter gill-raker space: the average distance between gill rakers from five measurements.

20 - Epibranchial gill-raker length: the average distance between the base of five gill rakers and their tip.

21 - Lower pharyngeal jaw (LPJ) width: Maximum external distance between the horns.

22 - Lower pharyngeal jaw (LPJ) length: Maximum distance from the imaginary midline between the caudal edge of the horns and the anteriormost tip of the plate.

23 - Lower pharyngeal jaw (LPJ) depth: Maximum distance between the dorsal surface of the plate and the ventral keel joining both toothplate bones.

Measurements taken on specimens used for genus-level comparisons presented in Figure 2. See Appendix 5 for details of each specimen.

1 - Standard Length (SL)

2 - Head length

3 - Head height

4 - Eye position

5 - Eye diameter

6 - Snout length, mouth closed

7 - Mouth position

8 - Body depth : measured vertically at the highest point of the body

9 – Caudal peduncle length, ventrally: straight distance between insertion of the last anal fin ray and the insertion of the ventral-most caudal fin ray into the hypural plate

10 – Caudal peduncle depth: vertically from dorsal to ventral edge of the peduncle at mid-length
