# Supplementary material for: Diet-Morphology Correlations in the Radiation of South American Geophagine Cichlids (Perciformes: Cichlidae: Cichlinae)
Source: PLoS One. 2012 Apr 2;7(4):e33997. doi: 10.1371/journal.pone.0033997 (PMC3317448; doi:10.1371/journal.pone.0033997)
Supplement: File S2 — Prey items included in each diet category. (DOC) [file pone.0033997.s005.doc]

**S2.** Prey items included in each diet category.

**Benthic prey:**

Copepoda, Diptera larvae, Misc microcrustacea, Amphipoda, Rotifers, Bivalvia, Diatoms, Nematodes, Horsehair worms, Oligochaeta, Other Annelida, Ostracoda, Seeds, Tardigrada, Collembolla.

**Vegetative detritus**

Vegetation detritus, Fine detritus, Unidentified debris, Woody debris

**Animal detritus**

Unidentified arthropod fragments, Scales

**Epibenthic prey**

Trichoptera, Turbellaria, Plecoptera, Ephemeroptera, Megaloptera, Hirudinea, Aquatic coleoptera adults, Aquatic coleoptera larvae, Briozoa, Lepidoptera larvae, Cladocera, Aquatic Hemiptera, Corixidae, Decapoda, Desmids, Eggs (fish), Eggs (invertebrates), Odonata nymphs, Hydracarina, Filamentous algae, Gastropoda

**Fish**

Anura (Indivual larvae), Fish, Fish bones, Fish fragments, Fish larvae

**Surface prey**

Arachnids, Diflugiids, Flowers, Misc. Terrestrial insects, Odonata adults, Orthoptera, Terrestrial Coleoptera, Terrestrial Hymenoptera, Terrestrial Hemiptera, *Wolffia* sp

**Water column prey**

Aquatic vegetation, Miscelaneous aquatic insects, *Chara* sp, Microscopic algae (blue/green), Sponges
